# Media matters: culture medium-dependent hypervariable phenotype of mesenchymal stromal cells

**DOI:** 10.1186/s13287-023-03589-w

**Published:** 2023-12-12

**Authors:** Joan C. Fitzgerald, Georgina Shaw, J. Mary Murphy, Frank Barry

**Affiliations:** Regenerative Medicine Institute (REMEDI), University of Galway, Galway, Ireland

**Keywords:** Mesenchymal stromal cells (MSCs), Culture medium, Surface proteome

## Abstract

**Background:**

Despite a long history of investigation and sustained efforts in clinical testing, the number of market authorisations for mesenchymal stromal cell (MSC) therapies remains limited, with none approved by the United States Food and Drug Administration. Several barriers are impeding the clinical progression of MSC therapies, to the forefront of these is a lack of standardised manufacturing protocols which is further compounded by an absence of biologically meaningful characterisation and release assays. A look at clinical trial registries demonstrates the diversity of MSC expansion protocols with variabilities in cell source, isolation method and expansion medium, among other culture variables, making it extraordinarily difficult to compare study outcomes. Current identification and characterisation standards are insufficient; they are not specific to MSCs and do not indicate cell function or therapeutic action.

**Methods:**

This work analysed the influence of five widely used culture media formulations on the colony-forming potential, proliferation kinetics, trilineage differentiation potential and immunomodulatory potential of human bone marrow-derived MSCs (BM-MSCs). The surface marker expression profiles were also characterised using a high-content flow cytometry screening panel of 243 markers.

**Results:**

Significant differences in the biological attributes of BM-MSCs including clonogenicity, proliferation, differentiation propensity and immunomodulatory capacity were revealed in response to the composition of the culture medium. Despite their biological differences, all cell preparations uniformly and strongly expressed the standard positive markers proposed for BM-MSCs: CD73, CD90 and CD105. Immunophenotypic profiling revealed that the culture medium also had a significant influence on the surface proteome, with one-third of tested markers exhibiting variable expression profiles. Principal component analysis demonstrated that BM-MSCs isolated and expanded in a proprietary xeno- and serum-free medium displayed the most consistent cell phenotypes with little variability between donors compared to platelet lysate and foetal bovine serum-containing media.

**Conclusions:**

These data suggest that media composition has a highly significant impact on the biological attributes of MSCs, but standard surface marker tests conceal these differences. The results indicate a need for (1) standardised approaches to manufacturing, with an essential focus on defined media and (2) new biologically relevant tests for MSC characterisation and product release.

**Supplementary Information:**

The online version contains supplementary material available at 10.1186/s13287-023-03589-w.

## Background

The field of cell therapy is a major frontier in medicine, promising regenerative and disease-modifying solutions for a host of significant diseases that represent unmet clinical need. The investigation of mesenchymal stromal cells (MSCs) has been to the forefront of this effort for more than 20 years. Clinical trial registries list hundreds of trials investigating MSCs in a broad range of diseases including orthopaedic, autoimmune, neurological, cardiovascular and retinal conditions. While the safety of MSC administration is broadly accepted, the efficacy of treatment in most conditions is unproven. This is reflected in a striking disproportionality between the number of registered MSC trials and the number of therapies receiving market authorisation. The majority of trials have been small-scale phase I and phase II studies, which by their design are limited by small patient numbers and lack of proper controls to establish the effect of the intervention [1]. The limited number of large-scale phase III trials indicates that there are significant barriers impeding the progression of MSC therapies along the clinical translation pipeline.

One such barrier, still unsurmounted, is the staggering array of variables in MSC manufacturing protocols. A look at published clinical trials administering MSCs highlights an extraordinary vulnerability of process, revealing variability in approach to every major process step including the tissue source, donor source (autologous or allogeneic), isolation procedure, expansion medium, harvesting and cryopreservation protocols, delivery vehicles and dose among many others. A great deal of attention has been paid to the impact of tissue source [2–5], donor heterogeneity [6, 7] and cryopreservation [8–10] on the phenotype of MSCs. In contrast it seems that the impact of medium composition on MSC heterogeneity is a fundamentally important problem, which has not been given necessary attention, particularly given that most conventional expansion media contain heterogeneous mixtures of unquantified and sometimes undefined components.

Conventional protocols have relied on foetal bovine serum (FBS) to provide the essential nutrients and growth factors for MSC isolation and expansion. However, this comes with risks of contamination of FBS with pathogens such as prions [11], endotoxin [12], adventitious viruses [13] and unidentified zoonoses and the potential for subsequent xenogeneic disease transmission. Further concerns of transplanted MSCs expanded in FBS eliciting severe immunological responses in patients to xenogeneic serum antigens [14–17] have led to the investigation of human-derived media supplements including human serum and more predominantly, human platelet lysate (PL). Though the use of products of human origin mitigates the xenogeneic concerns, risk of transmission of human disease remains an issue of concern [18, 19]. As heterogeneous and undefined products, both serum and PL are subject to inconsistent lot-to-lot performance, introducing considerable variability between experimental results. This makes it difficult to compare results between studies and complicates the interpretation of clinical trial results particularly when multiple small-scale trials are the main mode of assessment currently. In the case of PL, protocols for production at scale by pooling units from large donor pools have been developed to minimise the variation between batches [20–22]. However, prompted by fears of increased disease transmission risks, some regulatory bodies have recommended limiting donor pool sizes to 16 [23, 24]. Though patient safety must be prioritised, this move is likely to lead to greater inconsistency between batches with subsequent effects on the comparability and reproducibility of results. Many studies supplement these undefined supplements with additional growth factors such as epidermal growth factor (EGF), platelet-derived growth factor (PDGF)-BB and basic fibroblast growth factor (FGF2), introducing further process variability.

Given the limitations with human and animal-derived media components, there is a clear need for defined medium supplements which support reproducible manufacturing protocols for the production of consistent batches of MSCs. Serum-free and xeno-free media offer a more favourable solution compared to FBS and PL from a regulatory point of view, and there are several commercially available media for MSC expansion. However, the performance of some of these is uncertain and many do not support isolation from primary tissue. A novel xeno- and serum-free medium, Purstem 2 (PS2), previously developed in this laboratory, supports the isolation of MSCs from whole bone marrow and their subsequent expansion [25].

A number of previous studies have investigated the effect of culture media on the phenotype and functional attributes of MSCs. These have predominantly compared one selected media to the gold standard of 10% FBS. PL has been the most commonly studied supplement and has been the subject of many comparisons with FBS, the results of which have been analysed in recent systematic reviews [26–28], which concluded that there were no significant differences between the cells. Several groups have compared the performance of MSCs cultured in various commercially available serum-free media with mixed results [29–31]. Comprehensive studies comparing PS2 medium to standard FBS-containing medium both with and without additional supplementation with FGF2 for the isolation and expansion of MSCs have previously been performed demonstrating the superior performance of PS2 MSCs [25, 32]. Additional studies have assessed the effect of FGF2 addition to FBS media on cell phenotype and function [33–36]. Although the need for serum screening is widely acknowledged and is often performed in research and clinical settings, there are limited published data on the effect of different FBS batches on MSC phenotype. In general, studies assessing the effects of culture media on MSCs have focused on single comparisons and assess the minimal criteria for MSC identification only.

In this study, we have carried out a series of experiments to assess the impact of culture medium on the isolation, expansion and biological attributes of primary bone marrow-derived MSCs (BM-MSCs). We expanded cells from three bone marrow preparations, where all conditions were identical with the exception of the media composition. The impact of five different culture media were assessed, representative of those most commonly used, including (1) the PS2 proprietary xeno and serum-free supplement, (2) PL, an FBS batch which has been pre-screened for performance both (3) with and (4) without FGF2 supplementation and (5) an unscreened FBS batch with FGF2. Here we describe the impact of these media compositions on the phenotype and functional attributes of BM-MSCs including clonogenicity, proliferation kinetics, cell morphology, trilineage differentiation potential, immunosuppressive and immunogenic activities on T lymphocytes and surface immunophenotype. Surface marker expression profiles were also assessed using a high-content flow cytometry screening panel. The results indicate that changes in media composition dramatically alter the biological attributes of MSCs. However, this significant heterogeneity which undoubtedly impacts the therapeutic activity of the cells is undetected when standardised surface marker tests are used. The results suggest that (1) MSCs are hypervariable in culture and dependent upon the composition of the media and (2) current release tests fail completely to detect this variability. The work indicates that there is an urgent need to standardise the processing parameters employed for production of MSCs for clinical use, to remove from use the standard markers widely employed and to devise a new set of biological tests for product release.

## Methods

### Isolation and culture of BM-MSCs

Bone marrow aspirates (25–30 mL) were obtained from the iliac crest of three healthy volunteers (two male, one female) aged between 21 and 25 years at Galway University Hospital. Mononuclear cells (MNC) were counted and whole bone marrow was plated in five different culture media consisting of Alpha-MEM (Gibco) and 1% penicillin/streptomycin (10,000 units/mL, 10,000 µg/mL; Sigma) supplemented with:

- a proprietary serum/xeno- free medium formulation, PS2 [25]
- 5% Stemulate™ Pooled Human PL (Cook Regentec)
- 10% FBS selected from a serum screen of various FBS batches for suitability for MSC isolation and expansion (selected FBS; Hyclone)
- 10% selected FBS supplemented with 1 ng/mL of FGF2 (Peprotech)
- 10% FBS from a batch not selected from a serum screen (unselected FBS; Sigma) supplemented with 1 ng/mL FGF2.

MNCs were seeded at densities of 8.5 × 10^4^ MNCs/cm^2^ for both FBS + FGF2 conditions, 11.25 × 10^4^ MNCs/cm^2^ for PS2 and PL media and 22.5 × 10^4^ MNCs/cm^2^ for selected FBS alone. Cell cultures were incubated at 37 °C, 5% CO_2_. After 4 days, non-adherent cells were removed by washing the monolayers with D-PBS and fresh media were added. Media were replenished every 2–3 days until distinct colonies were evident when they were passaged. BM-MSCs were detached by incubation at 37 °C with TrypLE™ Express Enzyme 1x (Gibco) for up to 5 min and replated at a density of 3,000–5,000 cells/cm^2^. BM-MSCs were subcultured to passage 3, harvesting at 80–90% confluency and cells harvested from passage 3 were used for subsequent experiments. Excess cells were cryopreserved in 10% dimethyl sulfoxide (DMSO, Sigma) in human albumin solution (50 g/L, Baxter).

### Colony-forming unit-fibroblast (CFU-f) assay

To assess clonogenicity, 5 × 10^5^ MNCs were seeded in triplicate in 6-well plates with 3 mL of each culture medium. Media changes and washes were performed as described above. CFU-fs were fixed between days 8 and 10 in 95% ice cold methanol for 15 min, followed by staining with 2.3% crystal violet solution (Sigma) for 15 min. Clonogenicity was assessed by counting CFU-fs, defined as discrete colonies containing 30 cells or more.

### Cumulative population doublings

Cell growth rate was determined by calculating population doublings (PD) using the following formula:

$${\text{Population Doublings}} = \log {\text{ (cells harvested}}/{\text{cells }}\;{\text{seeded)}}/\log 2$$

Cumulative population doublings (CPD) were calculated by summing the PDs over time in culture. Population doubling time at each passage was calculated by dividing the time in culture in hours by the number of doublings which occurred in that time. The predicted cell yield at the end of passage 3 was calculated for each donor and media condition based on the calculated CFU-f formation and CPDs, assuming a 30 mL marrow aspirate was cultured in the relevant culture medium.

### Chondrogenic differentiation

Chondrogenic differentiation was assessed at passage 3 as described previously [37] with minor modifications. Briefly, 2 × 10^5^ BM-MSCs were placed in 1.5 mL Eppendorf tubes and centrifuged in a swing-out rotor at 100*g* for 5 min to form pellets and suspended in incomplete chondrogenic medium (ICM), consisting of DMEM high glucose (4.5 g/L) supplemented with 100 nM dexamethasone, 50 μg/mL ascorbic acid 2-P, 40 μg/mL L-proline, 1% ITS + Premix (6.25 μg/mL insulin, 6.25 μg/mL transferrin, 6.25 ng/mL selenous acid, 5.35 μg/mL linoleic acid and 1.25 mg/mL BSA), 1 mM sodium pyruvate and 100 units/mL penicillin, 100 µg/mL streptomycin. Control pellets were cultured in ICM throughout and test pellets received 10 ng/mL of transforming growth factor (TGF)-β3 (Peprotech). Pellets were maintained in culture at 37 °C, 5% CO_2_ and 2% O_2_ with medium changes every other day. Pellets were harvested after 21 days for sulphated glycosaminoglycan (GAG) quantitation (*n* = 3) and histologic evaluation (*n* = 1).

For histology, pellets were fixed in 10% neutral buffered formalin for 2 h and processed using a Leica ASP300S automatic tissue processor. Post-processing, pellets were embedded in paraffin wax and 5 µM sections were prepared and mounted on slides. Slides were deparaffinised in xylene and rehydrated by immersion in alcohol. To visualise sulphated GAG, slides were stained with 0.1% (w/v) Fast Green FCF and 0.1% (w/v) Safranin-O. Slides were mounted with DPX (Sigma) and imaged using an Olympus BX43 microscope.

For quantification of GAG, pellets were digested in 200 μL papain solution (2.5 μg/mL in 50 mM sodium phosphate, 2 mM N-acetyl cysteine, 2 mM ethylenediaminetetraacetic acid [EDTA], pH 6.5) at 60 °C for 16 h. GAG was measured by reaction with 1,9-dimethylmethylene blue [38]. DNA content of cell pellets were assessed using the Quant-iT Picogreen dsDNA assay kit (Molecular Probes) according to manufacturer’s instructions and GAG production per pellet was normalised to DNA content.

### Adipogenic differentiation

Adipogenic potential of BM-MSCs at passage 3 was assessed as described previously [39]. Lipid accumulation was visualised with Oil Red O staining and quantified using an adipogenesis quantification kit (Sigma) according to the manufacturer’s protocol. Lipid accumulation in each sample was normalised to DNA content as described previously.

### Osteogenic differentiation

Osteogenic propensity of BM-MSCs at passage 3 was assessed as described previously [39]. Calcium accumulation was visualised with Alizarin Red staining and quantified using Calcium LiquiColor™ test kit (Stanbio) according to manufacturer’s instructions. Calcium deposition in each sample was normalised to DNA content.

### Surface marker screening

Flow cytometry was performed to confirm cell identity in accordance with the International Society for Cell & Gene Therapy (ISCT) minimal criteria [40]. BM-MSCs at passage 3 were washed in FACS buffer (2% heat-inactivated FBS, 0.05% sodium azide in D-PBS), passed through a 40 µm cell strainer and blocked using BD Human Fc Block™ for 10 min before incubation for 30 min at 4 °C with the following R-phycoerythrin (PE)-conjugated antibodies: anti-CD73, anti-CD90, anti-CD105, anti-CD3, anti-CD14, anti-CD19, anti-CD34, anti-CD45, anti-HLA-DR and the appropriate isotype controls. Antibody details can be found in Additional file 1: Table S1. Samples were washed twice in FACS buffer and resuspended in 200 μL of FACS buffer for analysis. For dead cell discrimination, SYTOX™ Red (1:2000 dilution) was added 15 min before sample analysis. Samples were analysed on a FACS Canto II using FACS Diva software, and data analysis was performed using FlowJo™ software, version 10.8.1 (all BD Biosciences).

### Immune modulation assays

To assess whether BM-MSCs could suppress activated T lymphocyte proliferation, MSCs in culture at passage 3 were detached, washed in D-PBS and resuspended in co-culture medium consisting of RPMI 1640 (Sigma) supplemented with 10% heat-inactivated FBS (Hyclone), 100 units/mL penicillin, 100 µg/mL streptomycin, 2 mM L-glutamine, 0.1 mM non-essential amino acids, 1 mM sodium pyruvate (all Sigma) and 55 μM β-mercaptoethanol (Gibco). For each MSC preparation, 20,000, 5,000 and 2,000 MSCs (to create MSC:PBMC ratios of 1:5, 1:20 and 1:50, respectively) were added in duplicate to 96-well U-bottomed plates in 50 μL of co-culture medium and incubated at 37 °C, 5% CO_2_ for 2 h to allow attachment.

Peripheral blood mononuclear cells (PBMCs) were isolated from the blood of three healthy donors by density gradient centrifugation and stained with 5 μM CellTrace™ Violet (CTV; Invitrogen) according to manufacturer’s instructions. To each well of the 96-well U-bottomed plates, 1 × 10^5^ PBMCs in 50 μL of co-culture medium were added. T lymphocyte proliferation was stimulated by the addition of 50 μL of T lymphocyte activation medium per well, consisting of 0.05 μg/mL of Purified NA/LE Mouse Anti-Human CD3 (BD) and 10 μg/mL of Purified NA/LE Mouse Anti-Human CD28 (BD) in co-culture medium.

The immunogenicity of BM-MSC preparations was similarly assessed, except T lymphocytes were not stimulated with CD3/CD28 and received 50 μL of co-culture medium only. Furthermore, the assay was only performed at the MSC:PBMC ratio of 1:5. For both assays, each MSC preparation was assayed independently with three individual PBMC preparations from different biological donors.

After 4 days of incubation at 37 °C, 5% CO_2_, cells were washed in 200 μL of FACS buffer and subsequently incubated with the antibodies CD3-FITC, CD4-APC (both 1:40 dilution; Biolegend) for 30 min, protected from light. Cells were washed again with FACS buffer and stained with LIVE/DEAD™ Fixable Near-IR Dead Cell Stain Kit (Invitrogen; 1:4000 dilution) for 30 min, protected from light. After washing with FACS buffer, cells were fixed in 4% paraformaldehyde for 15 min at room temperature, washed again and resuspended in 200 μL of FACS buffer for analysis on a FACSCanto™ II cytometer using FACS Diva software. Data analysis was performed using FlowJo™ software. Percentage proliferation of T lymphocytes co-cultured with BM-MSCs was calculated relative to CD3/CD28 stimulated T lymphocyte controls and averaged across the three blood donors.

### High-content surface protein screening

The surface immunophenotypes of BM-MSC preparations were characterised using the BD Lyoplate™ Human Cell Surface Marker Screening Panel (BD Biosciences), containing 242 lyophilised monoclonal antibodies in 96-well format to specific cell surface proteins and the corresponding isotype controls [41]. Staining was performed as detailed in the manufacturer’s protocol with slight modifications. BM-MSCs were added at a density of 5 × 10^4^ cells per well to 96-well V-bottomed plates in 50 µL stain buffer (BD Pharmingen) containing 2 mM EDTA and stained with 10 μL of reconstituted antibody. Secondary Alexa Flour 647 conjugated antibodies were diluted at 1:400 for goat anti-mouse and 1:300 for goat anti-rat antibodies, respectively. A sample from each MSC preparation were also stained with an additional antibody, anti-CD317-PE (1:40 dilution). Following fixation in 4% PFA in D-PBS, cells were stored in the dark at 4 °C and analysed within 12 h.

### Surface protein data acquisition

Data acquisition was performed using a BD Accuri™ C6, BD FACSCanto II or Beckman Coulter CytoFLEX and 5000 events were collected for each sample. Analyses of FCS files were performed using C6 analysis software, BD FACSDiva™ software or FlowJo™ software version 10.8.1, respectively. The gating strategy is depicted in Fig. 6A. The per cent positive expression data for each sample are detailed in full in Additional file 2: Table S2.

### Surface marker data analysis

Markers were categorised based on their overall per cent positive expression levels across all 15 BM-MSC preparations (three donors cultured in five culture media): (a) ‘positive’: markers which displayed ≥ 85% expression in all samples regardless of culture condition or donor, (b) ‘negative’: ≤ 15% expression in all samples and (c) ‘variable’: markers with differences in percentage positive values between samples.

A heatmap of the 78 markers with variable expression profiles between samples was generated using ClustVis, a web tool for the visualisation of multivariate data [42]. No scaling was applied to rows, both rows and columns were clustered using correlation distance and average linkage.

A principal component analysis (PCA) of the total surface marker expression data (243 markers) was performed in ClustVis to illustrate the differences in surface marker expression profiles between MSCs cultured in different media. No scaling was applied to the data, singular value decomposition with imputation was used to calculate principal components.

### Statistical analysis

Statistical analyses were performed using GraphPad Prism^®^ Version 8.4.3 (GraphPad Software LLC). Data are presented as mean ± standard deviation (SD). Between-group comparisons were made using ordinary one-way or two-way ANOVA with Tukey’s post-test to account for multiple comparisons. In all experiments, a *p* value of < 0.05 was considered statistically significant.

## Results

### Isolation in PS2 results in colonies with a distinct morphology to isolation in FBS- and PL-containing media

**Fig. 1:**
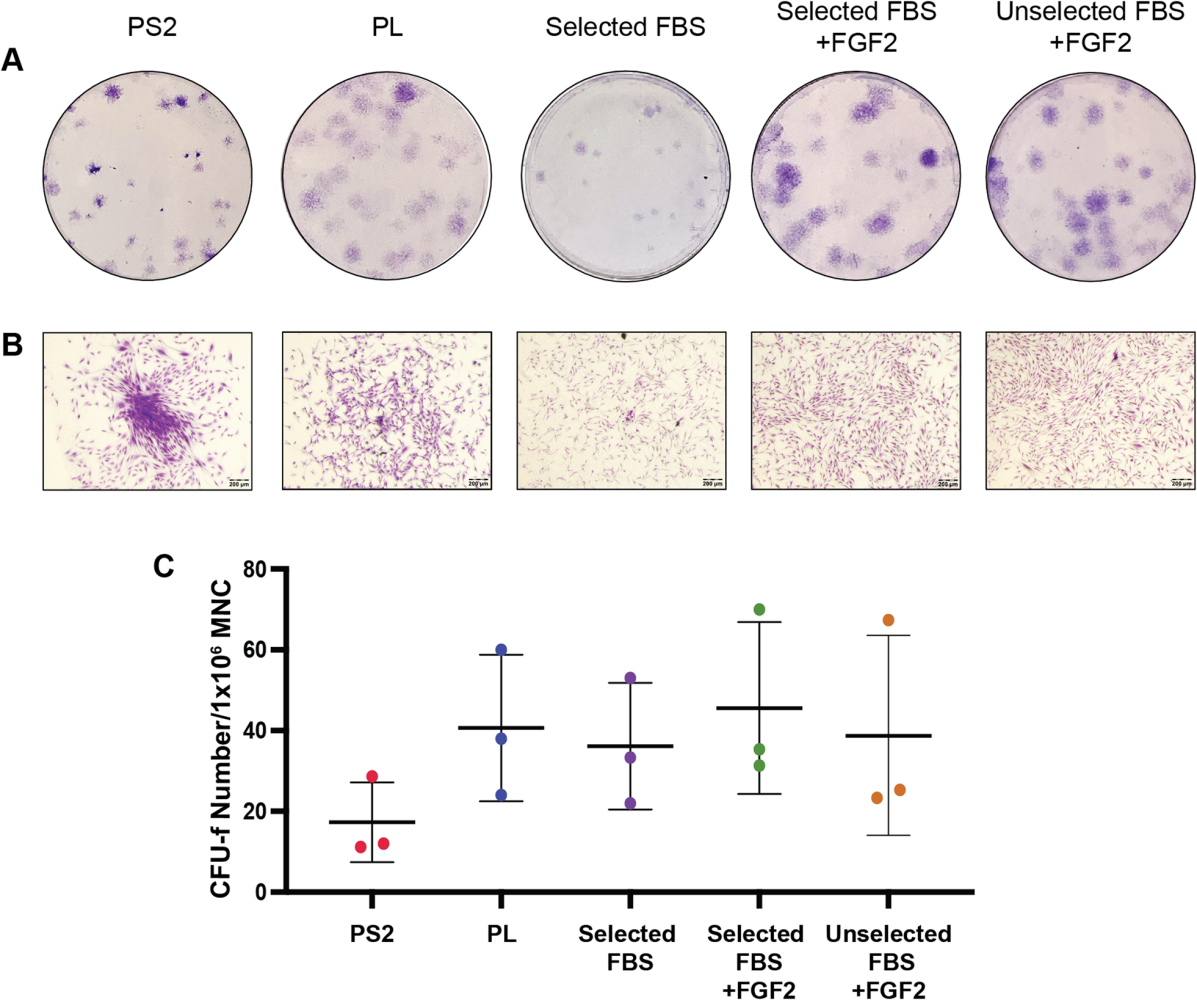
Clonogenicity of BM-MSCs isolated in different culture media. **A** Representative images of CFU-f wells stained with crystal violet 10 days after isolation from bone marrow. CFU-fs isolated in PS2 and 10% selected FBS alone medium appear smaller than those isolated in 5% PL and both selected and unselected FBS + FGF2. Colonies isolated in PS2 also appear more tightly clustered than those isolated in other media conditions. **B** Representative magnified images of colonies confirm that BM-MSCs isolated in PS2 form more dense colonies compared to those isolated in PL- and FBS-containing media which grew in larger, diffuse colonies. Scale bar = 200 μm. **C** Quantitative analysis of CFU-f number normalised per million MNC seeded. No statistically significant differences were observed between groups. Data points represent the mean of technical triplicates for each of three biological replicates (*n* = 3) with the error bars indicating standard deviation (SD)

Colonies isolated in PS2 medium displayed a distinct morphology, forming smaller, more dense clusters than those cultured in FBS + FGF2- and PL-containing media which grew in large diffuse colonies (Fig. 1A, B). Colony enumeration (Fig. 1C) demonstrated a trend towards fewer colonies isolated in the PS2 medium compared to all other groups which were broadly similar; however, differences were not statistically significant.

### Culture medium supplement has a significant impact on the proliferation rate of BM-MSCs

**Fig. 2:**
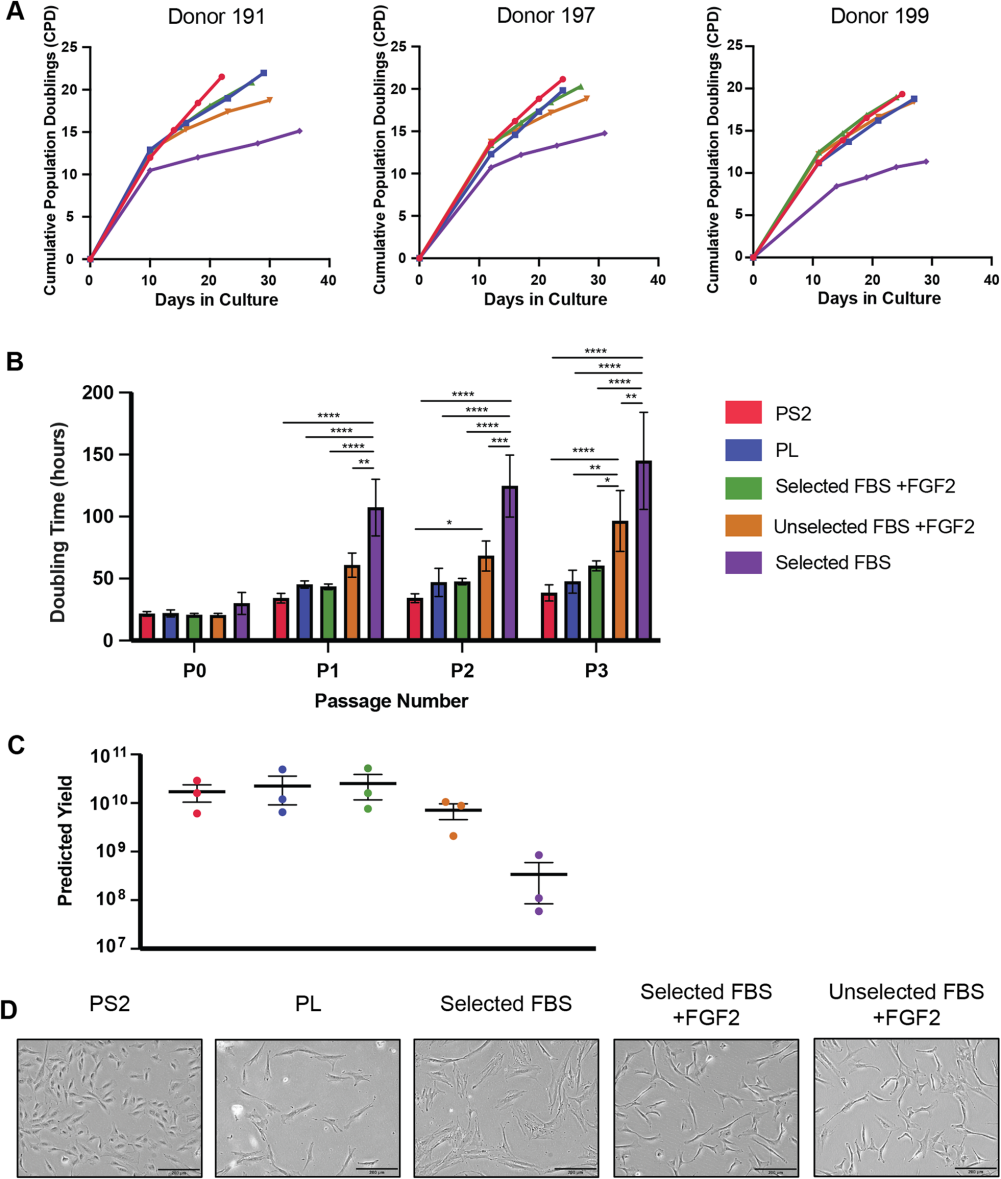
Culture medium has a significant impact on the proliferation rate and morphology of BM-MSCs. **A** Growth curves of BM-MSCs from three donors isolated and expanded up to passage 3 in five different culture media with results expressed as mean cumulative population doublings of technical triplicates over time in culture. **B** Doubling time at each passage with results expressed as mean ± SD of three biological replicates (*n* = 3) (*p ≤ 0.05, **p ≤ 0.01, ***p ≤ 0.001, ****p ≤ 0.0001). **C** Expected yield at end of passage 3 of three BM-MSC donors cultured in the five different culture media based on the CFU-f isolation and CPDs calculated, assuming a 30 mL marrow was cultured in the relevant group. There were no statistical differences observed in predicted yield between media groups. **D** Representative phase contrast images of BM-MSCs (Passage 3) indicate differences in morphology of BM-MSCs isolated and expanded in the various culture media. Scale bar = 200 μm

Growth kinetics of BM-MSCs to the end of passage 3 (Fig. 2A) demonstrated marked variations in proliferation rates, with MSCs in the 10% selected FBS alone group proliferating significantly slower than the other media groups. Analysis of the average doubling time per passage (Fig. 2B) revealed no differences in doubling time between conditions at passage 0; however, from passage 1 to passage 3, the doubling time for MSCs cultured in selected FBS alone was significantly higher than all other media conditions. MSCs cultured in unselected FBS + FGF2 displayed a longer doubling time than selected FBS + FGF2, with this difference increasing over time and was significantly higher than all other conditions (except selected FBS alone) at passage 3. Cells cultured in PS2 had the lowest doubling time across all three passages, but this was not significantly different to MSCs cultured in PL or in selected FBS + FGF2. Calculation of expected yield (Fig. 2C) demonstrated differences of up to three orders of magnitude between the most and least proliferative conditions. However, differences between culture conditions were not statistically significant.

### MSCs cultured in PS2 and in selected FBS alone have distinct morphologies compared to those cultured in FBS + FGF2 and PL

BM-MSCs isolated and expanded in PL and both selected and unselected FBS + FGF2 exhibited spindle-shaped, fibroblastic-like morphologies typical of MSCs (Fig. 2D). MSCs cultured in PS2 demonstrated a more rounded morphology and appeared smaller with fewer extended processes. In selected FBS alone, MSCs displayed more broadened, enlarged and flattened morphologies.

### The batch of FBS and the addition of FGF2 significantly impacts the chondrogenic propensity of BM-MSCs

**Fig. 3:**
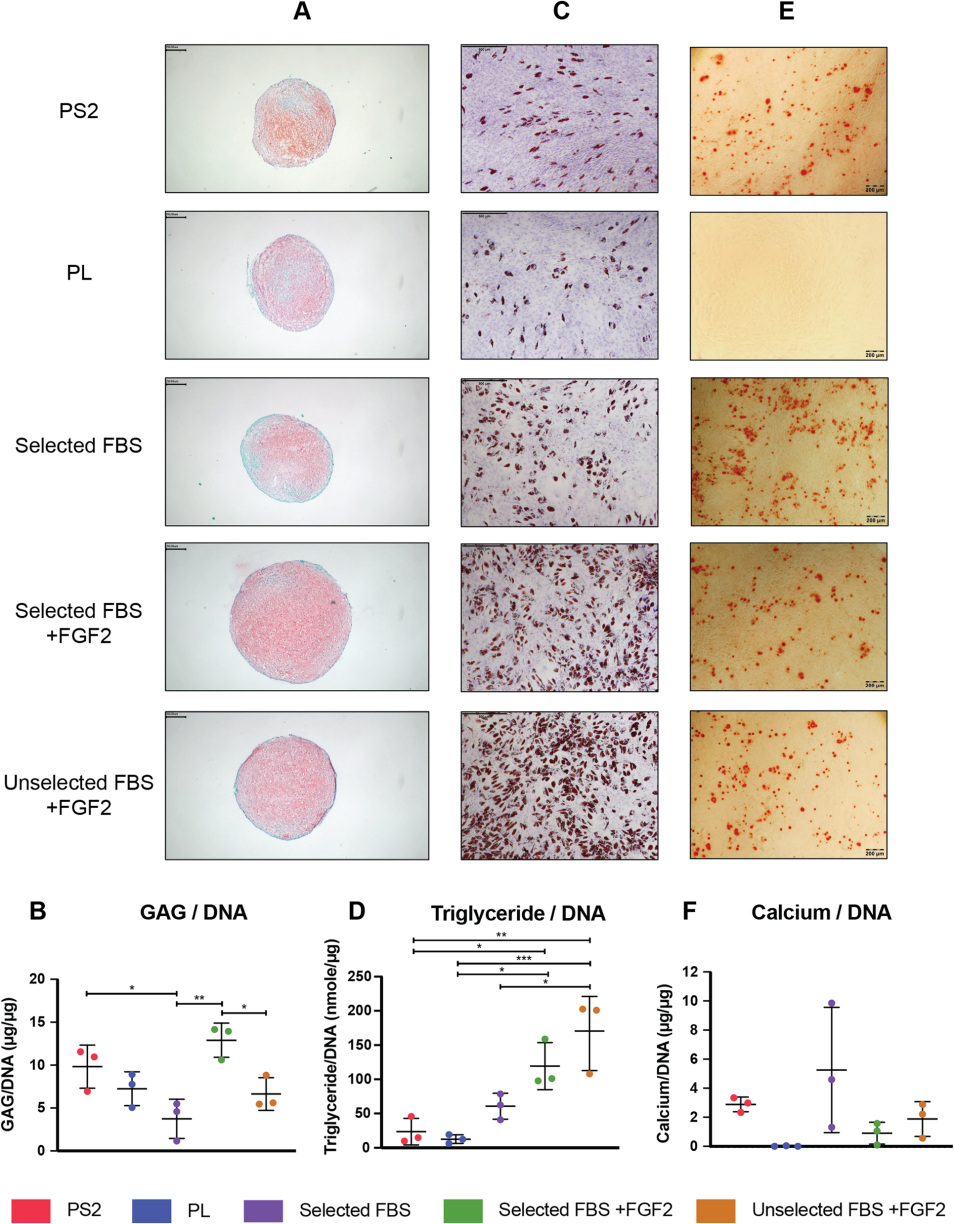
Trilineage differentiation propensities are significantly impacted by the composition of the culture medium. **A** Representative images of Safranin-O/Fastgreen staining of chondrogenically differentiated BM-MSCs. Scale bar = 250 μm. **B** Quantification of sulphated GAGs and normalisation to DNA content confirmed that chondrogenesis is significantly impacted by the batch of FBS used and the addition of FGF2 significantly increased chondrogenesis. Results are expressed as mean ± SD of three biological replicates (*n* = 3) (*p ≤ 0.05, **p ≤ 0.01). **C** Representative images of Oil Red O and H&E staining of adipogenically differentiated BM-MSCs demonstrates differences in the adipogenic capacity of BM-MSCs cultured in different media. Scale bar = 500 μm. **D** Quantification of triglyceride content and normalisation to DNA content confirms culture media formulation has a significant impact on the adipogenic propensity of the cells, with significantly increased adipogenesis occurring in FBS + FGF2 conditions compared to BM-MSCs cultured in PS2 and PL. Results are expressed as mean ± SD of three biological replicates (*n* = 3). **E** Representative images of Alizarin Red staining of osteogenically differentiated BM-MSCs demonstrates calcium deposition by MSCs cultured in all media conditions except PL. Scale bar = 200 μm. **F** Assessment of calcium quantification and normalisation to the DNA content confirmed lack of osteogenic differentiation in PL group. Calcium deposition by BM-MSCs cultured in the selected serum only group displayed large variability between donors. There were no statistically significant differences recorded between groups. Results are expressed as mean ± SD of three biological replicates (*n* = 3) (*p ≤ 0.05, **p ≤ 0.01, ***p ≤ 0.001)

Safranin-O/Fast Green FCF staining and quantification of sulphated GAGs with normalisation to DNA content (Fig. 3A, B) confirmed that chondrogenesis is significantly impacted by the batch of FBS; BM-MSCs cultured in selected FBS + FGF2 produced significantly higher GAG/DNA than those cultured in unselected FBS + FGF2. Furthermore, the addition of FGF2 significantly increased chondrogenesis with selected FBS + FGF2 producing significantly higher levels of GAG/DNA than selected FBS alone. After selected FBS + FGF2, cells cultured in PS2 medium were the next most chondrogenic when normalised to DNA content of the pellet, also producing significantly higher amounts of GAG/DNA than selected FBS alone. BM-MSCs cultured in PL displayed moderate chondrogenic propensity.

### Culture media supplement has a significant impact on the adipogenic potential of BM-MSCs

Oil Red O staining of lipid vacuoles revealed differences in lipid accumulation between BM-MSCs cultured in different media (Fig. 3C, D) which was confirmed by quantification of lipids and normalisation to DNA content. Statistical analysis demonstrated significant differences in adipogenic potential between media groups; both PS2 and PL groups demonstrated poor adipogenic potential and produced significantly lower levels of triglyceride/DNA than both FBS + FGF2 media groups. MSCs cultured in selected FBS alone were moderately adipogenic, but significantly lower than unselected FBS + FGF2, which had the highest lipid accumulation per cell.

### BM-MSCs isolated and expanded in all media displayed osteogenic potential except PL which did not undergo osteogenesis

Alizarin Red staining and quantification of calcium (Fig. 3E, F) demonstrated osteogenic propensity across all samples except in the case of MSCs cultured in PL, where there was no calcium detected by staining or quantification. Both FBS + FGF2-containing groups displayed limited calcium deposition across all three donors, whereas calcium accumulation per cell was slightly higher in the PS2 samples. The largest calcium accumulation was observed in the selected FBS alone group, but the range between biological donors was substantial and one particularly osteogenic donor in this medium may have skewed these results. Excepting the PL cultures, statistical analysis revealed no significant differences between groups.

### The immunosuppressive effects of BM-MSCs on activated T lymphocytes are dependent on the composition of the culture medium

**Fig. 4:**
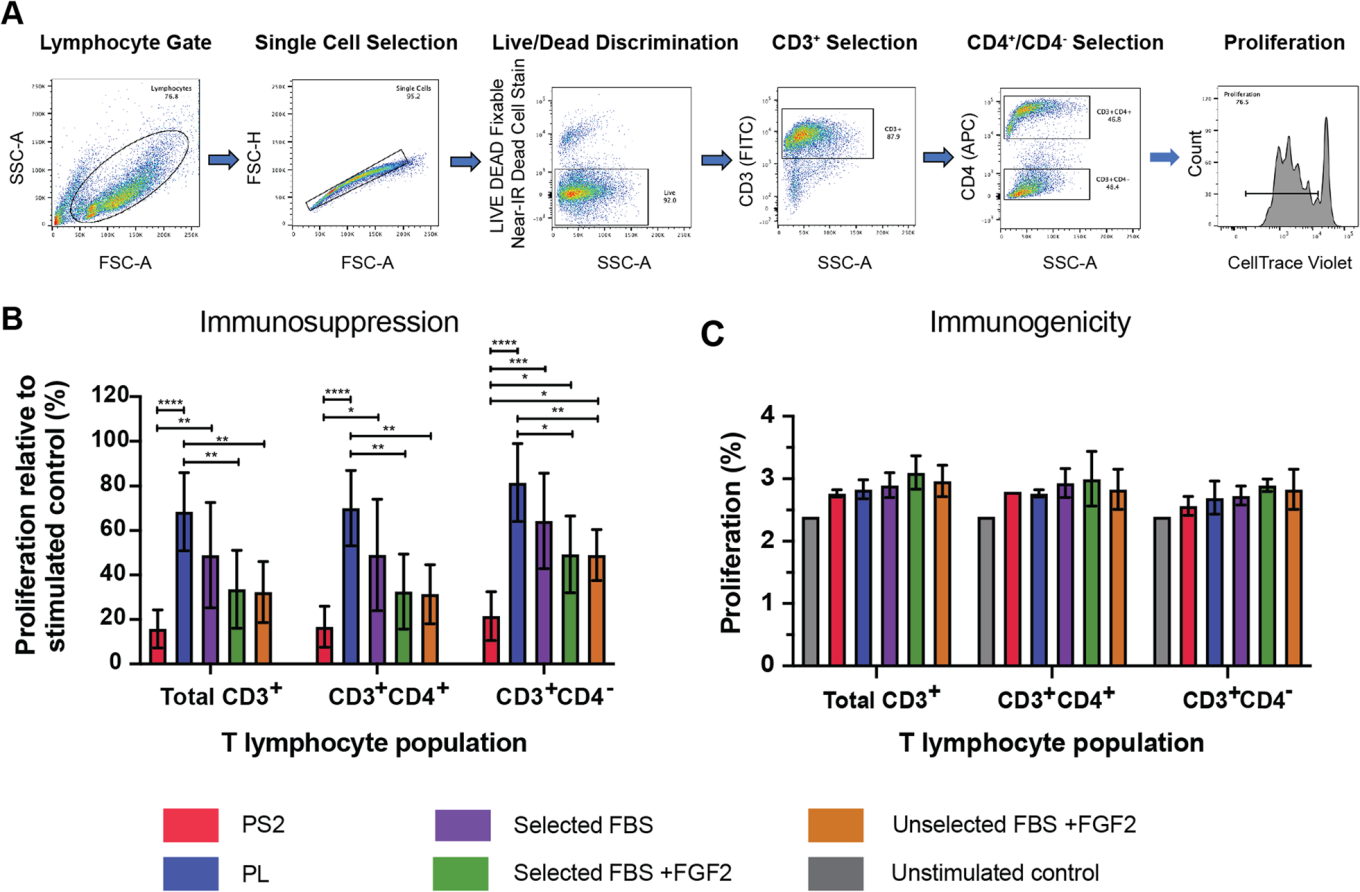
Immunosuppressive and immunogenic effects of BM-MSCs on T lymphocytes. **A** Representative gating strategy for flow cytometric analysis of T lymphocyte proliferation. **B** Immunosuppressive effects were assessed for each BM-MSC preparation on stimulated T lymphocytes isolated from the peripheral blood of three independent donors. BM-MSCs exerted significant immunosuppressive effects on stimulated T lymphocytes at a MSC:PBMC ratio of 1:5, and the size of these effects varied between culture conditions. No significant immunosuppressive effects were observed at higher ratios (data not shown). Results were expressed as the percentage proliferation relative to a CD3/CD28 stimulated T lymphocyte control averaged across the three blood donors. Bar graphs represent the mean ± SD of three biological replicates (*n* = 3) for each culture media condition (*p ≤ 0.05, **p ≤ 0.01, ***p ≤ 0.001, ****p ≤ 0.0001). **C** To assess potential immunogenic effects, percentage proliferation of unstimulated T lymphocytes co-cultured with BM-MSCs at a MSC:PBMC ratio of 1:5 for 4 days. Co-culture with BM-MSCs isolated and expanded in any media condition did not significantly increase T lymphocyte proliferation compared to unstimulated controls (grey bar). Results for each BM-MSC preparation were averaged across three blood donors. Bar graphs represent the mean ± SD of three biological replicates (*n* = 3) for each culture media condition

BM-MSCs exerted significant immunosuppressive effects on stimulated T lymphocytes at a MSC:PBMC ratio of 1:5, no significant immunosuppressive effects were observed at higher ratios of 1:20 and 1:50 (data not shown). MSCs cultured in PS2 exerted the strongest immunosuppressive effects in all three populations examined (total CD3^+^, CD3^+^CD4^+^ and CD3^+^CD4^−^). This was most significant in the CD3^+^CD4^−^ population, where PS2 MSCs were significantly more immunosuppressive than all other MSC groups. MSCs cultured in PL were the least immunosuppressive and were significantly less immunosuppressive than MSCs cultured in PS2 and both FBS + FGF2 groups across all three subsets (Fig. 4B). The immunosuppressive activity of both FBS + FGF2 groups were similar in all subsets; therefore, the selection of FBS batch had no effect on the immunosuppressive potential of BM-MSCs in this study. Cells cultured in selected FBS alone displayed reduced immunosuppressive capacity over FGF2-containing conditions, although this difference was not statistically significant. MSCs from this group, however, were significantly less immunosuppressive than PS2 MSCs across all T lymphocyte subsets examined.

### BM-MSCs cultured in all culture media conditions do not exert immunogenic effects on unstimulated T lymphocytes

Co-culture with BM-MSCs isolated and expanded in any media condition did not significantly increase T lymphocyte proliferation compared to unstimulated controls in any of the T lymphocyte subset groups (Fig. 4C). There was a slight increase in T lymphocyte proliferation in all PBMC groups which were co-cultured with BM-MSCs, the highest of which was observed in FBS-containing media and in particular those cultured with FGF2. However, there were no significant differences between groups.

### BM-MSCs cultured in all media conditions display the typical surface immunophenotype, except for HLA-DR which was elevated in FBS + FGF2 conditions

**Fig. 5:**
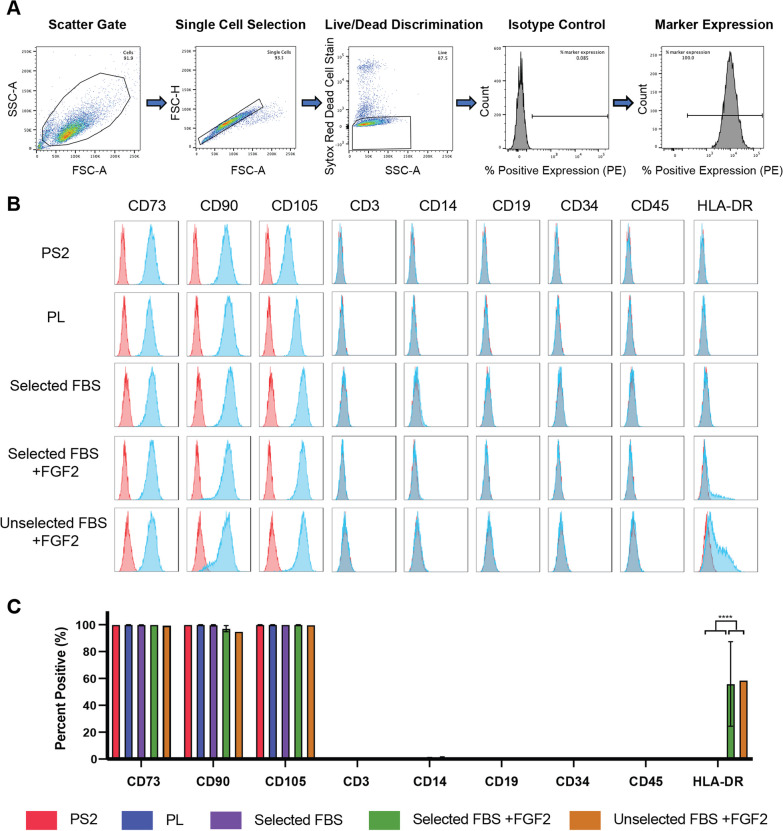
Surface marker characterisation of BM-MSCs using the standard ISCT panel of markers. **A** Representative gating strategy for flow cytometric analysis of BM-MSC surface marker expression. **B** Representative surface marker profile analysis of BM-MSCs at passage 3 for each culture condition. Blue histograms represent each antigen with red overlays representing the corresponding isotype control. **C** Bar graphs indicate the mean percentage of positive expression for each surface marker. BM-MSCs cultured in all conditions expressed the standard positive surface markers (≥ 90% expression of CD73, CD90, CD105) and lacked expression (≤ 2%) of all negative markers except HLA-DR the expression of which was significantly upregulated in both FBS + FGF2 conditions. Results are presented as the mean ± SD of three biological replicates (*n* = 3) (****p ≤ 0.0001)

Expression of the ISCT-proposed panel of surface markers for BM-MSC identification were assessed by flow cytometry (Fig. 5). BM-MSCs cultured in all conditions expressed the standard positive surface phenotype (≥ 90% expression of CD73, CD90, CD105) and lacked expression (≤ 2%) of the negative markers CD3, CD14, CD19, CD34 and CD45. There were marked variations in the expression of HLA-DR, a phenotypic marker of antigen presenting cells and proposed negative marker of BM-MSCs. The expression of this protein was significantly higher in both FBS + FGF2 conditions (range 19.8–77.6% expression) compared to the other three media groups which displayed < 2% expression in all samples.

### Cluster and principal component analyses of surface marker expression profiles revealed BM-MSCs cultured in PS2 demonstrated the least inter-donor heterogeneity in surface immunophenotypes

**Fig. 6:**
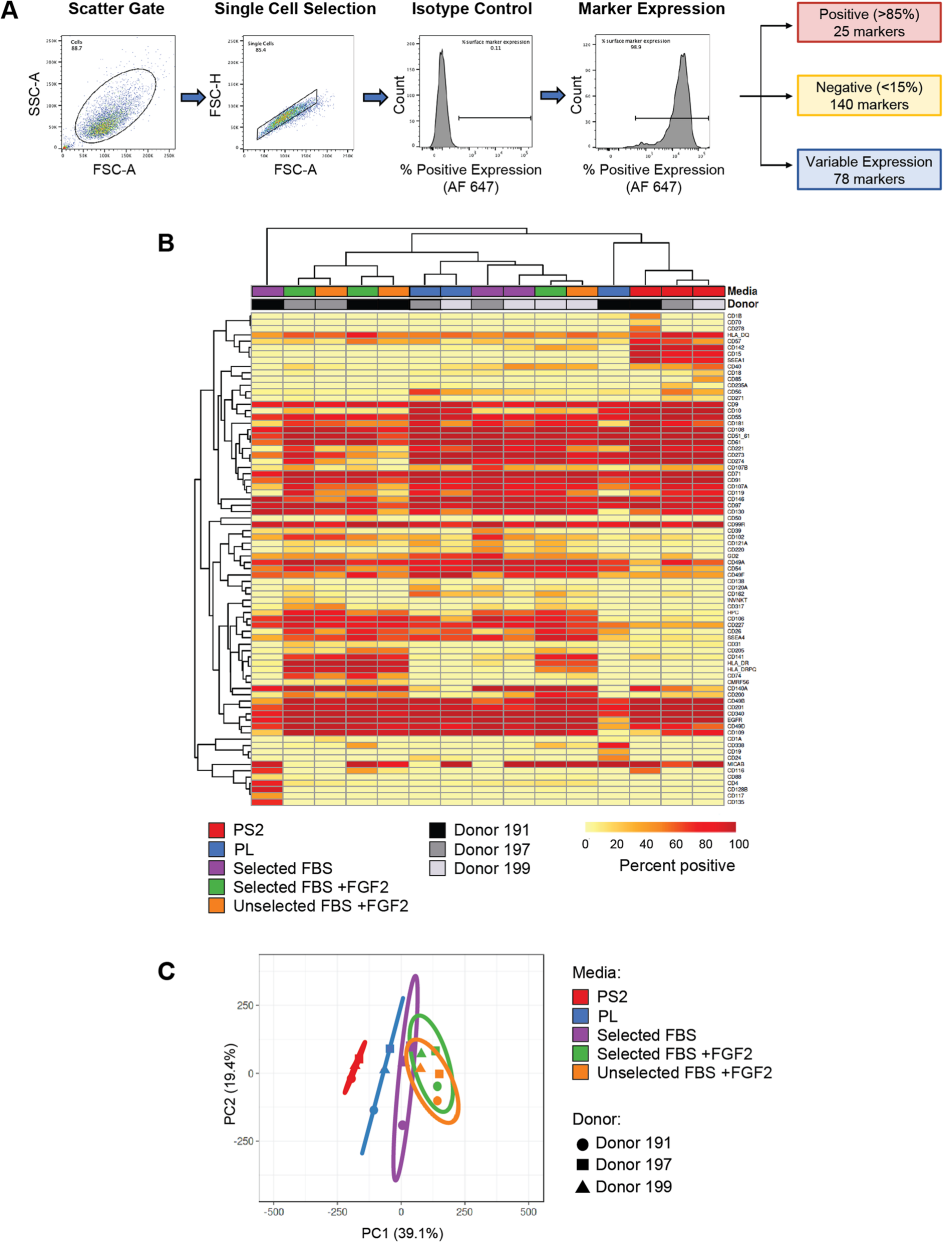
BM-MSCs cultured in PS2 demonstrated the most consistent surface marker expression profiles. **A** Representative gating strategy for BD Lyoplate™ flow cytometric analysis. Surface markers were then categorised according to their expression level across all samples. **B** Heatmap showing per cent positive expression of the 78 markers (rows) which displayed variable expression levels among samples (columns). No scaling was applied to rows, both rows and columns were clustered using correlation distance and average linkage. Cluster analysis demonstrates considerable variability due to both donor and culture media, except in the case of PS2 where MSCs from all three donors clustered together, indicating similarity in surface marker expression. **C** Principal component analysis on the global surface immunophenotype confirms less variability in surface marker expression profile when BM-MSCs are isolated and expanded in PS2 medium. No scaling was applied to rows; SVD with imputation was used to calculate principal components. The *X* and *Y* axes show principal component 1 and principal component 2 that explain 39.1% and 19.4% of the total variance, respectively. Prediction ellipses are such that with probability 0.95, a new observation from the same group will fall inside the ellipse. Media conditions and individual donors are displayed (*n* = 15 data points). PCA segregated MSCs cultured in PS2 and PL into distinct clusters which were separate from MSCs cultured in FBS-containing medium. MSCs cultured in PS2 displayed the lowest variability with all samples clustering tightly together

Markers were categorised according to their expression levels across all samples (Fig. 6A). Of the 243 surface proteins assessed, 25 markers were uniformly positive with per cent positive values of > 85% in all donors and culture conditions. Over half of all markers assessed—140—were negative (< 15%) in all samples and 78 surface markers displayed variable expression levels between BM-MSC preparations. The ISCT-proposed positive markers for BM-MSCs: CD73, CD90 and CD105, were contained in the group of 25 surface markers which were positive (> 85% positive expression) in all MSC preparations, along with other commonly used MSC markers CD13, CD29, CD44 and CD166. The per cent positive expression data for each sample are detailed in Additional file 1: Table S2.

To identify potential patterns in surface marker expression levels, a heatmap was generated and cluster analysis performed for the 78 markers which displayed variable expression levels between conditions was performed using ClustVis (Fig. 6B). Both columns (samples) and rows (surface markers) were ordered using distance-based clustering and average linkage. BM-MSCs isolated from all three donors and cultured in PS2 medium demonstrated the most consistent surface marker expression profiles demonstrated by these three samples clustering closest together. This was not replicated in any other media group, in all other samples along with variability in marker expression between culture conditions, donor variability was also a considerable factor. In the selected and unselected FBS + FGF2 conditions, these clustered more closely by donor than by culture condition.

PCA of all 243 markers was performed in ClustVis to assess the differences in global surface marker expression profiles between the MSC preparations (Fig. 6C). The prediction ellipses—indicating the probability with 95% confidence that new observations from the same group will fall inside the ellipse—for selected FBS + FGF2 and unselected FBS + FGF2 were almost coincident, confirming a high degree of similarity between these samples. Marker expression profiles in these samples displayed a degree of similarity with the MSCs cultured in selected FBS alone. Variability between donors in all three media groups was considerable, indicated by the spread of the individual donors and the size of the prediction ellipses. PCA segregated MSCs cultured in PS2 and those cultured in PL into two distinct clusters which were also distinct from MSCs cultured in all FBS-containing media. In addition, MSCs cultured in PS2 displayed the lowest variability between donors with all samples clustering closely together compared to other media conditions, suggesting that MSCs cultured in PS2 have a more homogenous phenotype than those cultured in undefined media containing FBS and PL.

## Discussion

Despite sustained efforts in clinical testing, progression to market of MSC therapies is yet to meet expectation, with mixed clinical results and few market approvals [43]. Central to this lack of progression is that the term ‘MSC’ is an ill-defined concept and in a clinical setting is a misnomer. Classical MSCs, first conceptualised by Friedenstein, have been defined as ‘postnatal, self-renewing, and multipotent stem cells giving rise to all the skeletal tissues’ [44]. However, what we have been attempting to apply clinically is a heterogeneous culture of stromal cells—within which may exist a subpopulation of ‘classical MSCs’—established by plastic adherence from bulk bone marrow, adipose tissue, umbilical cord or virtually any other connective tissue which we then expand using diverse protocols and undefined culture media, introducing further heterogeneity. Secondly, a set of weak standards for in vitro characterisation to these cultures has been applied which are (1) not specific to MSCs or indeed any type of cell and (2) not biologically relevant or linked to a specific mechanism of action. Finally, these cells are delivered through various routes of administration to patients in attempts to treat an enormous range of diverse conditions without a strong biological basis or understanding of the mechanism of action. It is not surprising therefore that although over 1000 MSC-based clinical trials have been initiated, the number of clinical approvals is less than 1% of that number.

This work primarily focused on one of the major variables in MSC manufacturing protocols: culture medium and its effect on the phenotypic and functional attributes of MSCs. While other studies have primarily focused on subsets of these media comparisons and usually single comparisons, this work presents a comprehensive and quantitative parallel analysis of the effect of five of the most commonly used culture media supplements on the basic and traditional in vitro hallmarks of BM-MSCs. The results presented here indicate that, when all other variables remain constant, the composition of the expansion medium has a dramatic impact on the biological attributes of the cells. MSCs, isolated from the same donor bone marrow were processed under identical conditions with the exception of the culture media. Differences in CFU-F formation (Fig. 1), morphology and proliferative activity (Fig. 2) were evident and the significant impact of the inclusion of FGF2 was also clear. It appeared that the inclusion of this growth factor had greater impact than the performance of a serum screen, suggesting that it alone may account for this difference. Although the xeno-free PS2 medium supported growth of cells with lower CFU-F activity, these formed denser colonies, possibly indicating a greater degree of homogeneity.

A capacity for trilineage differentiation was evident in cells expanded in all media (Fig. 3) with the exception of the PL-supplemented formulation. In this case, adipogenic differentiation of the cells was lowest and osteogenic activity was absent. Furthermore, the selection of FBS batch proved to have a significant effect on differentiation, even with the addition of FGF2. While it is understood that trilineage differentiation is not a key therapeutic mechanism in many applications of MSCs, and is rarely employed as a release criterion [45], this observation points to considerable biological variability in MSC production, which is a source of concern.

Immunomodulation is considered to be a central aspect of MSC therapeutic function and we observed substantial differences in the capacity of the cells to modulate the activity of stimulated T lymphocytes (Fig. 4). Immunosuppressive activity was highest in cells expanded in the xeno-free PS2 medium and lowest in cells expanded in PL-supplemented media. This raises quite serious concerns given the increasing use of PL as a substitute for FBS [20, 46]. Recent systematic reviews of studies comparing PL and FBS for MSC expansion have reported no significant differences in immunosuppression [27, 28]; however, the differences observed in this study, particularly between PL and PS2, are stark. One potential reason for enhanced immunosuppression in MSCs cultured in PS2 could be that this effect is extracellular vesicle (EV)-mediated. Palama et al. report an increase in EV production when BM-MSCs are cultured in PS2 medium compared to those cultured in 10% FBS + FGF2 [32] and similar increases in EV production when cells are cultured in commercial low-serum media compared to 10% FBS have been observed in other cell types [47]. EVs have been shown to inhibit the proliferation of lymphocytes [48], but cell–cell contact may still be required for optimal immunomodulatory effects [49].

Despite the biological differences observed here, all BM-MSC preparations met the minimal criteria for surface marker expression as defined by the ISCT [40], except for HLA-DR the expression of which was elevated in all MSC conditions cultured in 10% FBS with FGF2 (range 19.8% to 77.6% expression). The induction of HLA-DR expression in MSCs cultured in FBS with FGF2 has been well documented in the literature [35, 50, 51], with increased HLA-DR expression observed with higher concentrations of FGF2 [35]. HLA-DR expression is induced in proliferating MSCs by the binding of FGF2 to the FGF receptor, activating the MAPK/ERK-1/2 signalling pathway which controls the induction of the class II MHC transcription activator (CIITA) protein [50]. Although not observed here, other studies have reported elevated HLA-DR expression in clinical batches of MSCs expanded in PL [52]. TGFβ1 is a known inhibitor of CIITA [50], and the relative concentrations of TGFβ1 in PL and FBS may explain why HLA-DR expression was not induced in MSCs cultured in PL in this study [27].

Increased HLA-DR expression did not affect the immunosuppressive capacity of the MSC preparations on activated T lymphocytes, and similar to a previous report [35], suppression of T lymphocyte proliferation was in fact enhanced when co-cultured with MSCs cultured in FBS with FGF2, compared to FBS alone. Similarly, there was no significant effect on the immunogenic potential of MSCs regardless of HLA-DR expression levels, likely because T lymphocyte co-stimulatory molecules CD80 and CD86 were not expressed. Similar to trilineage differentiation, very few clinical protocols report HLA-DR expression and it rarely is employed as a release criterion [45, 53, 54].

The three positive surface markers, CD73, CD90 and CD105, were consistently expressed by BM-MSCs isolated and cultured in all media formulations. Despite this, we have quantified clear biological differences between MSCs cultured in different growth media and this must be reflected by differences in their surface proteome. Therefore, these markers fail to reveal or describe the differences in growth kinetics, morphology, trilineage differentiation and immunomodulatory potential described in these experiments. In the absence of proper standards and well-defined, understood characterisation assays, these minimal criteria are being loosely and inconsistently applied as release criteria. There is a clear and urgent need for new informative surface markers or alternative tests which strengthen release criteria.

The BD Lyoplate™ analysis (Additional file 2: Table S2) revealed several surface markers which were differentially expressed between cells expanded in the different media groups, some of which may reflect diverse biological attributes. SSEA-1 (stage-specific embryonic antigen-1, CD15), an embryonic marker, was highly expressed (> 75% positive) in cells isolated and expanded in PS2, but negative in all other groups, suggesting that isolation and culture in PS2 results in a more primitive cell phenotype. Several integrins associated with cell adhesion including CD49a, CD49d and CD49f were downregulated in BM-MSCs cultured in PS2, which may explain the smaller and less elongated morphology observed in these cells compared to those cultured in FBS and PL.

However, it could be the case that there is no definitive surface marker which defines MSC identity or function. The solution to improved release standards may reside in devising assays based on disease-specific mechanisms of action, generated from in vivo studies involving the transplantation and retrieval of labelled cells. Given that MSCs are proposed to exert broad therapeutic effects, release testing should consist of a matrix of tests including a range of immune assays and analysis of the paracrine secretome including soluble factors and extracellular vesicles. These rigorous testing protocols, though costly and time-consuming would be viable in allogeneic manufacturing models. Given the extent of variation in the biological properties of MSCs in response to the culture medium, this work supports the adoption of a unified approach to MSC manufacturing. Standardising manufacturing protocols and in particular media formulations could go some way to elucidating therapeutic mechanisms and improving interpretation of clinical results by removing one confounding variable and allowing reasonable comparison between studies.

The PCA and hierarchical cluster analyses (Fig. 6) revealed culture medium specific classification in the PS2 samples, these samples clustered closely together and distinctly from all others. This suggests that culture in PS2 results in more homogenous MSC preparations with more reproducible immunophenotypes. Samples from all other medium groups were more widely dispersed, indicating donor variation is also a significant factor in the surface marker expression profile of BM-MSCs cultured in PL and FBS. This is an interesting observation. PS2 is a relatively defined medium with far fewer constituents compared to FBS or PL; therefore, it is logical to accept that the resulting BM-MSC preparations are associated with greater homogeneity.

This raises the interesting question of whether the greater homogeneity in surface proteome associated with PS2 cultured MSCs (and inversely the heterogeneity in surface proteome with FBS and PL cultured cells) is acquired during expansion and/or if the media select different subpopulations of progenitors from the marrow. Although not statistically significant, we observed a trend towards lower CFU-f formation in MSCs isolated in PS2 compared with other media supplements (Fig. 1) which could indicate that this medium selects a smaller subset of MSC progenitors from the bone marrow than FBS and PL supplements. Previous studies comparing MSCs cultured in PL and FBS concluded that the selected populations were not inherently different and the biological differences were culture induced and reversible [26]. However, unlike these culture supplements, PS2 contains a small number of mostly defined components.

From the hierarchical clustering and PCA, it is clear that MSCs cultured in PS2 demonstrate the most consistent surface marker expression profiles and display less heterogeneity between donors than MSCs cultured in PL and FBS. This highlights the advantages of using defined media and provides further evidence to support a move away from undefined media supplements such as sera and PL towards serum-free and chemically defined culture media to reduce MSC heterogeneity.

## Conclusions

Serious concerns arise because of broad inconsistencies in the manufacture of MSC products, which have been accepted in an unchecked manner. If we accept the principle that ‘the process is the product’ then the term ‘MSC therapy’ encompasses a multitude of diverse cell products which bear little resemblance to each other. Current clinical protocols have been adapted directly from research laboratories, resulting in a staggering array of approaches. In particular, expansion protocols have relied on using undefined, heterogeneous and batch variable medium supplements which risk transforming the biological phenotype of the cells and must have a major bearing on the clinical outcomes.

The extensive heterogeneity introduced during the manufacturing process emphasises the need for unambiguous tests of product identity and therapeutic efficacy. However, a set of minimal identification criteria, relating to specific surface markers, differentiation propensity and culture characteristics, have been widely adopted in release testing. Despite their almost universal application, these tests are exceptionally poor at defining important biological characteristics of cells for clinical application. Rather than reveal, these tests conceal the profound biological variability in cells prepared under different conditions. The purpose of a release test of a medicinal product is to uncover batch differences and provide the manufacturer with a sensitive test that will reveal product inconsistencies. The purpose is also to provide regulators and quality specialists with a set of tools that will ensure that an identical product is released for patient use. The current ‘gold’ standards, promulgated by the ISCT in 2006, completely fail in this regard. While the intention was to set rigorous standards for MSC production, the outcome has been the exact opposite and attention to new guidelines for MSC characterisation and release testing is urgently needed.

It appears that with the deficiency of current characterisation protocols, until we standardise manufacturing methods we cannot be confident of consistency of cell products. These data demonstrate significant variation in the biological properties of MSCs in response to the culture medium which may necessitate the adoption of a unified approach to MSC manufacturing. This study has also provided some interesting insights into the impact of culture medium on the surface proteome of BM-MSCs and highlights the advantages of used defined media in terms of consistency of phenotype.   It provides further evidence to support a move away from undefined media supplements such as sera and PL towards serum-free and chemically defined culture media to reduce MSC heterogeneity.

### Supplementary Information

**Additional file 1. Supplementary Information 1:** Antibody details for flow cytometric characterisation of BM-MSC surface immunophenotype.

**Additional file 2. Supplementary Information 2:** Surface marker expression data (percentage of positive expression) for each individual marker, culture condition and donor (D191, D197 and D199 respectively), as assessed by BD Lyoplate^TM^ Human Cell Surface Marker Screening Panel.

## Data Availability

All data generated or analysed during this study are included in this published article and its supplementary information files.

## Abbreviations

BM: Bone marrow
CFU-f: Colony-forming unit-fibroblastic
CPD: Cumulative population doublings
CTV: CellTrace™ Violet
D-PBS: Dulbecco's phosphate-buffered saline
EDTA: Ethylenediamine tetra acetic acid
EGF: Epidermal growth factor
EV: Extracellular vesicle
FBS: Foetal bovine serum
FGF: Fibroblast growth factor
GAG: Glycosaminoglycan
ICM: Incomplete chondrogenic medium
ISCT: International Society for Cell & Gene Therapy
MNC: Mononuclear cell
MSC: Mesenchymal stromal cell
PBMC: Peripheral blood mononuclear cell
PCA: Principal component analysis
PD: Population doublings
PDGF: Platelet-derived growth factor
PL: Platelet lysate
PS2: Purstem 2 serum-free medium supplement
SD: Standard deviation
SSEA: Stage-specific embryonic antigen
TGF: Transforming growth factor

## References

[1] Kabat M, Bobkov I, Kumar S, Grumet M (2020). Trends in mesenchymal stem cell clinical trials 2004–2018: is efficacy optimal in a narrow dose range?. Stem Cells Transl Med.

[2] Binch ALA, Richardson SM, Hoyland JA, Barry FP (2019). Combinatorial conditioning of adipose derived-mesenchymal stem cells enhances their neurovascular potential: implications for intervertebral disc degeneration. JOR Spine.

[3] Xu L, Liu Y, Sun Y, Wang B, Xiong Y, Lin W (2017). Tissue source determines the differentiation potentials of mesenchymal stem cells: a comparative study of human mesenchymal stem cells from bone marrow and adipose tissue. Stem Cell Res Ther.

[4] Bortolotti F, Ukovich L, Razban V, Martinelli V, Ruozi G, Pelos B (2015). In vivo therapeutic potential of mesenchymal stromal cells depends on the source and the isolation procedure. Stem Cell Rep.

[5] Torensma R, Prins HJ, Schrama E, Verwiel ET, Martens AC, Roelofs H (2013). The impact of cell source, culture methodology, culture location, and individual donors on gene expression profiles of bone marrow-derived and adipose-derived stromal cells. Stem Cells Dev.

[6] Siegel G, Kluba T, Hermanutz-Klein U, Bieback K, Northoff H, Schäfer R (2013). Phenotype, donor age and gender affect function of human bone marrow-derived mesenchymal stromal cells. BMC Med.

[7] Phinney DG, Kopen G, Righter W, Webster S, Tremain N, Prockop DJ (1999). Donor variation in the growth properties and osteogenic potential of human marrow stromal cells. J Cell Biochem.

[8] Francois M, Copland IB, Yuan S, Romieu-Mourez R, Waller EK, Galipeau J (2012). Cryopreserved mesenchymal stromal cells display impaired immunosuppressive properties as a result of heat-shock response and impaired interferon-gamma licensing. Cytotherapy.

[9] Pollock K, Sumstad D, Kadidlo D, McKenna DH, Hubel A (2015). Clinical mesenchymal stromal cell products undergo functional changes in response to freezing. Cytotherapy.

[10] Antebi B, Asher AM, Rodriguez LA, Moore RK, Mohammadipoor A, Cancio LC (2019). Cryopreserved mesenchymal stem cells regain functional potency following a 24-h acclimation period. J Transl Med.

[11] Chou ML, Bailey A, Avory T, Tanimoto J, Burnouf T (2015). Removal of transmissible spongiform encephalopathy prion from large volumes of cell culture media supplemented with fetal bovine serum by using hollow fiber anion-exchange membrane chromatography. PLoS ONE.

[12] Kirikae T, Tamura H, Hashizume M, Kirikae F, Uemura Y, Tanaka S (1997). Endotoxin contamination in fetal bovine serum and its influence on tumor necrosis factor production by macrophage-like cells J774.1 cultured in the presence of the serum. Int J Immunopharmacol.

[13] Hawkes PW (2015). Fetal bovine serum: geographic origin and regulatory relevance of viral contamination. Bioresour Bioprocess.

[14] Horwitz EM, Gordon PL, Koo WK, Marx JC, Neel MD, McNall RY (2002). Isolated allogeneic bone marrow-derived mesenchymal cells engraft and stimulate growth in children with osteogenesis imperfecta: implications for cell therapy of bone. Proc Natl Acad Sci USA.

[15] Sundin M, Ringdén O, Sundberg B, Nava S, Götherström C, Le Blanc K (2007). No alloantibodies against mesenchymal stromal cells, but presence of anti-fetal calf serum antibodies, after transplantation in allogeneic hematopoietic stem cell recipients. Haematologica.

[16] Selvaggi TA, Walker RE, Fleisher TA (1997). Development of antibodies to fetal calf serum with arthus-like reactions in human immunodeficiency virus-infected patients given syngeneic lymphocyte infusions. Blood.

[17] Mackensen A, Dräger R, Schlesier M, Mertelsmann R, Lindemann A (2000). Presence of IgE antibodies to bovine serum albumin in a patient developing anaphylaxis after vaccination with human peptide-pulsed dendritic cells. Cancer Immunol Immunother.

[18] Iudicone P, Fioravanti D, Bonanno G, Miceli M, Lavorino C, Totta P (2014). Pathogen-free, plasma-poor platelet lysate and expansion of human mesenchymal stem cells. J Transl Med.

[19] Hemeda H, Giebel B, Wagner W (2014). Evaluation of human platelet lysate versus fetal bovine serum for culture of mesenchymal stromal cells. Cytotherapy.

[20] Schallmoser K, Bartmann C, Rohde E, Reinisch A, Kashofer K, Stadelmeyer E (2007). Human platelet lysate can replace fetal bovine serum for clinical-scale expansion of functional mesenchymal stromal cells. Transfusion.

[21] Schallmoser K, Strunk D (2009). Preparation of pooled human platelet lysate (pHPL) as an efficient supplement for animal serum-free human stem cell cultures. J Vis Exp JoVE.

[22] Schallmoser K, Strunk D, Helgason CD, Miller CL (2013). Generation of a pool of human platelet lysate and efficient use in cell culture. Basic cell culture protocols.

[23] Stuhler A, Blumel J (2015). Specific aspects for virus safety of raw materials for cellular-based medicinal products. Bundesgesundheitsblatt Gesundheitsforschung Gesundheitsschutz.

[24] Bieback K, Fernandez-Munoz B, Pati S, Schafer R (2019). Gaps in the knowledge of human platelet lysate as a cell culture supplement for cell therapy: a joint publication from the AABB and the International Society for Cell & Gene Therapy. Cytotherapy.

[25] Barry FP, Mooney EJ, Murphy JM, Shaw GM, Gaynard SP, inventors. Serum-free medium. 2015. 20.08.2015.

[26] Fernandez-Rebollo E, Mentrup B, Ebert R, Franzen J, Abagnale G, Sieben T (2017). Human platelet lysate versus fetal calf serum: these supplements do not select for different mesenchymal stromal cells. Sci Rep.

[27] Guiotto M, Raffoul W, Hart AM, Riehle MO, di Summa PG (2020). Human platelet lysate to substitute fetal bovine serum in hMSC expansion for translational applications: a systematic review. J Transl Med.

[28] Palombella S, Perucca Orfei C, Castellini G, Gianola S, Lopa S, Mastrogiacomo M (2022). Systematic review and meta-analysis on the use of human platelet lysate for mesenchymal stem cell cultures: comparison with fetal bovine serum and considerations on the production protocol. Stem Cell Res Ther.

[29] Lee JY, Kang MH, Jang JE, Lee JE, Yang Y, Choi JY (2022). Comparative analysis of mesenchymal stem cells cultivated in serum free media. Sci Rep.

[30] Bhat S, Viswanathan P, Chandanala S, Prasanna SJ, Seetharam RN (2021). Expansion and characterization of bone marrow derived human mesenchymal stromal cells in serum-free conditions. Sci Rep.

[31] Bobis-Wozowicz S, Kmiotek K, Kania K, Karnas E, Labedz-Maslowska A, Sekula M (2017). Diverse impact of xeno-free conditions on biological and regenerative properties of hUC-MSCs and their extracellular vesicles. J Mol Med (Berl).

[32] Palama MEF, Shaw GM, Carluccio S, Reverberi D, Sercia L, Persano L (2020). The secretome derived from mesenchymal stromal cells cultured in a xeno-free medium promotes human cartilage recovery in vitro. Front Bioeng Biotechnol.

[33] Bianchi G, Banfi A, Mastrogiacomo M, Notaro R, Luzzatto L, Cancedda R (2003). Ex vivo enrichment of mesenchymal cell progenitors by fibroblast growth factor 2. Exp Cell Res.

[34] Solchaga LA, Penick K, Porter JD, Goldberg VM, Caplan AI, Welter JF (2005). FGF-2 enhances the mitotic and chondrogenic potentials of human adult bone marrow-derived mesenchymal stem cells. J Cell Physiol.

[35] Sotiropoulou PA, Perez SA, Salagianni M, Baxevanis CN, Papamichail M (2006). Characterization of the optimal culture conditions for clinical scale production of human mesenchymal stem cells. Stem Cells.

[36] Hagmann S, Moradi B, Frank S, Dreher T, Kammerer PW, Richter W (2013). FGF-2 addition during expansion of human bone marrow-derived stromal cells alters MSC surface marker distribution and chondrogenic differentiation potential. Cell Prolif.

[37] Murphy JM, Dixon K, Beck S, Fabian D, Feldman A, Barry F (2002). Reduced chondrogenic and adipogenic activity of mesenchymal stem cells from patients with advanced osteoarthritis. Arthritis Rheum.

[38] Farndale RW, Sayers CA, Barrett AJ (1982). A direct spectrophotometric microassay for sulfated glycosaminoglycans in cartilage cultures. Connect Tissue Res.

[39] Fitzgerald JC, Duffy N, Cattaruzzi G, Vitrani F, Paulitti A, Mazzarol F (2022). GMP-compliant production of autologous adipose-derived stromal cells in the NANT 001 closed automated bioreactor. Front Bioeng Biotechnol.

[40] Dominici M, Le Blanc K, Mueller I, Slaper-Cortenbach I, Marini F, Krause D (2006). Minimal criteria for defining multipotent mesenchymal stromal cells. The International Society for Cellular Therapy position statement. Cytotherapy.

[41] BD Biosciences. BD Lyoplate™ screening panels human cell surface markers/mouse cell surface markers. 2013. https://www.bdbiosciences.com/content/dam/bdb/marketing-documents/BD_Lyoplate_Screen_Panels.pdf.

[42] Metsalu T, Vilo J (2015). ClustVis: a web tool for visualizing clustering of multivariate data using Principal Component Analysis and heatmap. Nucleic Acids Res.

[43] Trounson A, McDonald C (2015). Stem cell therapies in clinical trials: progress and challenges. Cell Stem Cell.

[44] Bianco P (2014). "Mesenchymal" stem cells. Annu Rev Cell Dev Biol.

[45] Wilson AJ, Rand E, Webster AJ, Genever PG (2021). Characterisation of mesenchymal stromal cells in clinical trial reports: analysis of published descriptors. Stem Cell Res Ther.

[46] Burnouf T, Strunk D, Koh MB, Schallmoser K (2016). Human platelet lysate: Replacing fetal bovine serum as a gold standard for human cell propagation?. Biomaterials.

[47] Li J, Lee Y, Johansson HJ, Mäger I, Vader P, Nordin JZ (2015). Serum-free culture alters the quantity and protein composition of neuroblastoma-derived extracellular vesicles. J Extracell Vesicles.

[48] Mokarizadeh A, Delirezh N, Morshedi A, Mosayebi G, Farshid AA, Mardani K (2012). Microvesicles derived from mesenchymal stem cells: potent organelles for induction of tolerogenic signaling. Immunol Lett.

[49] Conforti A, Scarsella M, Starc N, Giorda E, Biagini S, Proia A (2014). Microvescicles derived from mesenchymal stromal cells are not as effective as their cellular counterpart in the ability to modulate immune responses in vitro. Stem Cells Dev.

[50] Bocelli-Tyndall C, Zajac P, Di Maggio N, Trella E, Benvenuto F, Iezzi G (2010). Fibroblast growth factor 2 and platelet-derived growth factor, but not platelet lysate, induce proliferation-dependent, functional class II major histocompatibility complex antigen in human mesenchymal stem cells. Arthritis Rheum.

[51] Tarte K, Gaillard J, Lataillade JJ, Fouillard L, Becker M, Mossafa H (2010). Clinical-grade production of human mesenchymal stromal cells: occurrence of aneuploidy without transformation. Blood.

[52] Grau-Vorster M, Laitinen A, Nystedt J, Vives J (2019). HLA-DR expression in clinical-grade bone marrow-derived multipotent mesenchymal stromal cells: a two-site study. Stem Cell Res Ther.

[53] Trento C, Bernardo ME, Nagler A, Kuci S, Bornhauser M, Kohl U (2018). Manufacturing mesenchymal stromal cells for the treatment of graft-versus-host disease: a survey among centers affiliated with the european society for blood and marrow transplantation. Biol Blood Marrow Transplant.

[54] Mendicino M, Bailey AM, Wonnacott K, Puri RK, Bauer SR (2014). MSC-based product characterization for clinical trials: an FDA perspective. Cell Stem Cell.

