# Supplementary material for: Media matters: culture medium-dependent hypervariable phenotype of mesenchymal stromal cells
Source: Stem Cell Res Ther. 2023 Dec 12;14:363. doi: 10.1186/s13287-023-03589-w (PMC10717324; doi:10.1186/s13287-023-03589-w)
Supplement: Supplementary file 1 — Additional file 1. Supplementary Information 1: Antibody details for flow cytometric characterisation of BM-MSC surface immunophenotype. [file 13287_2023_3589_MOESM1_ESM.pdf]

Supplementary Information 1:
Antibody details for flow cytometric characterisation of BM-MSC surface immunophenotype.

Antigen	Supplier	Catalogue Number	Dilution
CD105	Invitrogen	MHCD10504	1:50
CD73	BD	550257	1:25
CD90	BD	555596	1:500
CD14	AbD Serotec	SFL2185	1:50
CD19	BD	345777	1:25
CD3	BD	345765	1:25
CD34	BD	555822	1:25
CD45	BD	555483	1:25
HLA-DR	Invitrogen	MHLDR04	1:25
IgG1κ isotype	BD	554680	
IgG2b isotype	Invitrogen	MG2B04	
