# Supplementary material for: Media matters: culture medium-dependent hypervariable phenotype of mesenchymal stromal cells
Source: Stem Cell Res Ther. 2023 Dec 12;14:363. doi: 10.1186/s13287-023-03589-w (PMC10717324; doi:10.1186/s13287-023-03589-w)
Supplement: Supplementary file 2 — Additional file 2. Supplementary Information 2: Surface marker expression data (percentage of positive expression) for each individual marker, culture condition and donor (D191, D197 and D199 respectively), as assessed by BD LyoplateTM Human Cell Surface Marker Screening Panel. [file 13287_2023_3589_MOESM2_ESM.pdf]

Supplementary Information 2:
Surface marker expression data (percentage of positive expression) for each individual marker, culture condition and donor (D191, D197 and D199 respectively), as assessed by BD Lyoplate™ Human Cell Surface Marker Screening Panel.

Marker	PS2			PL			Selected FBS			Selected FBS +FGF2			Unselected FBS +FGF2		
	D191	D197	D199	D191	D197	D199	D191	D197	D199	D191	D197	D199	D191	D197	D199
CD1a	1.63	0.5	0.4	6.28	2.7	0.6	0.07	0.3	1.4	1.4	0.7	1.6	0.09	15.4	1.3
CD1b	51.8	0.6	0.7	10.5	3.1	1	0.36	0.6	1.3	0.23	0.8	1.7	0.16	0.12	1.5
CD1d	0.57	0.5	0.3	0.39	2.3	0.8	0.12	0.3	1.4	0.21	0.6	1.4	0.09	0.22	1.4
CD2	0	0.2	0.3	0.31	2.1	0.7	0.07	0.3	1	0.26	0.7	1.1	0.14	0.24	0.8
CD3	0.17	0.5	0.5	0.41	2.6	0.9	0.1	0.4	1	0.4	0.4	1.3	0.16	0.22	1.4
CD4	0.9	1	1.3	1	3.6	5.1	73.6	9.5	11.4	6.38	12.1	14.9	3.2	9.19	12
CD4v4	0.8	1	1.9	0.81	2.3	3	2.1	6.8	6.2	3.89	7.1	8.9	1.94	4.68	6.6
CD5	0.17	0.7	0.1	0.44	1.4	1	0.16	0.5	1.1	0.24	0.5	1.1	0.29	0.28	1.3
CD6	0.1	0.3	0.6	0.44	1.8	0.7	0.017	0.5	1	0.14	0.4	1.1	0.19	0.18	0.8
CD7	0.07	1.9	1	0.37	1.3	0.9	0.14	0.4	1.1	0.25	0.8	1.2	0.17	0	0.7
CD8a	0.2	0.3	0.4	0.38	1	0.7	0.1	0.2	1.1	0.26	0.3	1.3	0.14	0.32	0.9
CD8b	0.14	0.7	0.6	0.5	2.6	1.1	0.19	0.6	1.3	0.19	0.6	1.6	0.24	0.4	1.2
CD9	92.1	96.8	95.9	90	98.4	96.8	73.5	72.9	81.2	92.7	88.4	91.8	85.6	78.9	90.7
CD10	99.8	99.6	99.4	75.5	96.3	91.8	0.7	15.7	12	9.34	30.6	31.9	9.05	9	23.3
CD11a	1.22	0.4	0.2	0.31	1.7	0.9	0.14	0.2	1	0.16	0.5	1.2	0.07	0.28	1
CD11b	0.43	0.3	0.6	0.26	1.5	1.2	0.17	0.3	0.9	0.22	0.8	1.3	0.17	0.3	1.4
CD11c	0.03	0.3	0.1	0.37	1.9	0.8	0.1	0.5	0.9	0.29	0.3	0.8	0.17	0.22	1
CD13	98.3	99.9	100	98.8	100	99.9	97.7	99.9	99.9	99.3	99.9	99.8	96.6	99.5	99.9
CD14	0.83	2	0.4	1.39	1.1	1	2.24	0.4	1.1	1.17	0.4	1.2	2.83	0.3	2.4
CD15	96	83.1	79.4	0.42	1.5	0.9	3.86	0.5	1	0.26	0.6	0.8	0.18	0.28	1
CD15s	0.23	0.2	0.6	0.4	0.7	0.8	0.1	0.4	0.5	0.28	0.4	1	0.15	0.26	1.2
CD16	0.16	0.3	1.7	0.24	1.1	0.6	11.8	0.2	0.6	0.22	0.3	1	0.1	0.12	0.9
CD18	0.33	8	22	1.93	7.5	9.7	0.48	10	5.3	2.54	7	6.7	0.19	0.32	3.3

Marker	PS2			PL			Selected FBS			Selected FBS +FGF2			Unselected FBS +FGF2		
	D191	D197	D199	D191	D197	D199	D191	D197	D199	D191	D197	D199	D191	D197	D199
CD19	0.07	0.2	0.3	34.3	1.4	0.9	0.03	0.5	0.9	0.26	0.7	1.1	0.14	0.26	1.2
CD20	0.13	0.5	0.9	0.46	1.4	0.9	0.28	0.5	1.6	1.1	0.7	1.1	0.09	0.2	1.9
CD21	0.23	0.1	0.6	0.28	1.2	0.9	0.05	0.3	1.4	0.2	4.2	0.8	0.1	0.37	1.1
CD22	0.2	0.3	0.4	0.37	1.1	0.9	0.03	0.4	1.2	0.14	0.8	1.1	0.05	0.28	1
CD23	0.07	0.2	0.3	0.35	1.3	0.8	0.12	0.3	1	0.17	0.5	1	0.05	0.26	1
CD24	0.17	0.4	1.2	50.3	18.7	8.2	2.26	1	6.1	0.31	0.6	1.2	0.27	0.16	1.6
CD25	0.07	0.2	0.4	4.48	1.1	0.7	4.44	0.4	1.2	0.19	0.4	1.3	2.42	0.16	1.1
CD26	1.16	6.2	1.6	38.2	50.3	63	5.06	14.4	42.6	73.3	70.9	76.1	62.3	37.1	65.3
CD27	0.17	0.5	0.6	0.16	1.6	0.9	6.31	0.3	1.3	0.17	0.5	0.8	1.98	0.12	1.1
CD28	0.1	0.2	0.4	0.14	1	0.7	0.27	0.5	1.1	0.21	0.4	1	0.18	0.26	1
CD29	99.8	100	100	98.3	99.9	99.9	95.6	100	100	98.8	100	99.9	96.5	99.5	99.9
CD30	0.43	0.5	0.7	1.7	1.7	1.3	1.6	0.8	0.7	1.64	0.9	1.8	2.12	0.26	0.9
CD31	6.28	6.9	8.7	0.41	4	1.1	0.15	2.5	3.3	15.8	24.8	7.8	11.4	12.9	7.5
CD32	0.13	0.3	0.4	0.29	1.7	0.9	0.07	0.6	1.4	0.7	0.9	1.2	0.1	0.2	1
CD33	0.03	0.2	0.4	0.23	1.8	0.9	0.02	0.5	1.2	8.91	1.4	1	0.08	0.2	1.4
CD34	1.59	3	2.3	0.45	1.4	0.8	0.03	0.6	1.6	0.28	0.9	1.6	0.2	0.47	1.7
CD35	0.2	0.3	0.3	0.32	1.5	0.6	0.02	0.3	1.2	0.17	0.5	1	0.1	0.31	0.9
CD36	0.13	0.5	0.4	0.34	1.4	1.2	0.28	0.7	1.2	0.31	0.6	0.9	0.39	0.2	1.6
CD37	0.3	0.5	0.3	0.27	1.5	0.7	0.02	0.4	1	0.12	0.6	0.9	0.1	0.2	1.1
CD38	0.1	0.3	12.9	0.32	1.1	0.8	0.24	6.2	4.4	0.57	4.6	4	0.27	1.46	2
CD39	0.17	0.5	0.8	0.47	0.9	1.2	14.9	47.7	17.9	2.04	21.8	4.1	4.84	23.1	3.2
CD40	33.6	38.6	57.9	0.48	3.3	13.8	2.25	18.1	45.8	3.17	17.5	37.3	3.8	8.54	32.7
CD41a	0.1	0.4	0.4	0.48	1.2	2.9	0.13	0.6	0.9	0.22	0.4	1	0.16	0.2	0.8
CD41b	2.05	0.2	0.5	0.29	1.3	1.3	0.17	0.5	1.3	0.19	0.7	1.3	0.1	0.06	0.9
CD42a	0.1	0.9	1.2	1.89	2.4	2.2	2.09	1.4	1.9	0.31	1.6	2.9	0.62	0.9	3.3
CD42b	0.1	0.4	0.5	0.3	2	0.7	0.14	0.5	1.4	1.1	0.8	1.2	0.08	0.28	0.9

Marker	PS2			PL			Selected FBS			Selected FBS +FGF2			Unselected FBS +FGF2		
	D191	D197	D199	D191	D197	D199	D191	D197	D199	D191	D197	D199	D191	D197	D199
CD43	0.43	0.8	1.2	0.39	3.3	1.9	0.09	1.2	1.7	0.17	0.9	1.2	0.15	0.26	1
CD44	98.4	99.9	100	99.2	100	99.8	98.1	99.6	99.6	98.9	98.5	99.5	100	94.6	99.2
CD45	0.99	0.4	0.7	1.31	1.5	1.3	0.07	0.5	1.1	0.27	0.6	1.2	0.44	0.32	1
CD45RA	0.03	0.5	0.7	0.41	1.1	0.9	0.31	0.5	1.1	0.27	0.8	1.2	0.05	0.22	1.7
CD45RB	0.3	0.7	0.4	0.25	1.3	0.8	0.07	0.5	0.9	0.24	0.5	1.1	0.08	0.22	1
CD45RO	0.23	0.5	0.9	1.1	1.2	1.3	0.87	0.4	1.2	0.19	0.7	1.3	0.27	0.37	1.3
CD46	97.9	100	99.9	98.5	100	100	97.9	100	100	100	100	100	99.1	99.7	99.9
CD47	99.9	100	100	99.9	100	100	99.9	100	100	99.9	99.9	100	99.9	99.6	100
CD48	1.25	0.4	0.4	0.43	1.6	0.8	1.63	0.5	1.3	1.74	0.5	1.1	1.56	0.26	1
CD49a	27.4	82	59.3	66.5	87.1	84.1	71.8	99.4	94.9	71.5	99.5	96.6	77.2	97.4	95.7
CD49b	98.7	99.3	99.7	69	98.9	97.7	37.9	57	89.6	99.9	99.1	99.8	98	96.3	99.7
CD49c	99.9	99.9	100	99.4	100	100	98.3	99.9	99.9	99.9	99.9	100	98.4	99.7	99.9
CD49d	77.9	76	58	34	94.6	84.5	68.5	98.5	94.5	97	99.5	99.2	89.2	98.6	97.5
CD49e	99.8	99.9	100	99.1	99.9	100	99.2	99.8	99.9	99.9	99.9	99.9	99.6	99.6	99.8
CD50	2.45	0.8	0.6	1.03	2.3	1.3	0.31	4.5	2.8	19.3	2.4	2.7	2.57	0.47	2
CD51/ CD61	97	100	99.9	98.2	99.9	99.9	67	99.9	96.4	97.8	99.7	97.6	64.2	97.6	92.8
CD53	0.13	0.3	0.6	1.05	0.8	0.6	1.63	0.4	1.2	1.28	0.6	0.9	1.04	0.24	1
CD54	11.2	39	30.1	66.6	77.6	74	51.2	91.1	90.7	41.7	73.8	81.1	45.7	62.6	81.9
CD55	94.9	99.4	96.7	87.4	98.7	94.3	68.4	85	85.4	78.2	88.8	83	68.7	80.6	77.8
CD56	9.75	54	32	12.2	67.3	37.6	6.78	26.7	21.4	6.83	8.5	13.2	5.21	5.39	8.8
CD57	85.6	66	40.6	12.4	38.1	9	16.3	25.8	14.5	53.6	24.9	6.1	32.2	10.7	15
CD58	99.5	100	100	99.4	100	100	98.7	100	100	99.1	100	100	98.8	99.6	100
CD59	99.9	99.8	100	99.9	99.9	99.8	100	99.7	99.9	100	99.9	99.9	100	99.8	99.8
CD61	99.7	100	99.8	99.6	100	99.8	56.9	99.9	93.6	99.5	99.6	96.2	66.9	95.9	90.7
CD62E	2.74	0.4	0.3	0.48	1.8	0.8	0.91	0.4	1	0.16	0.5	1.3	0.12	0.22	1.2
CD62L	0.23	0.3	0.6	0.45	1.6	0.7	0.05	0.5	1.1	0.36	0.7	1.2	0.13	0.25	1.1

Marker	PS2			PL			Selected FBS			Selected FBS +FGF2			Unselected FBS +FGF2		
	D191	D197	D199	D191	D197	D199	D191	D197	D199	D191	D197	D199	D191	D197	D199
CD62P	0.1	0.4	0.4	0.25	1.6	0.9	0.13	0.5	1.4	0.17	0.9	1.1	0.1	0.14	0.9
CD63	98.5	99.9	100	98.1	100	99.9	98.9	99.8	99.8	97.3	99.9	99.9	96.6	99.7	99.8
CD64	2.02	0.4	0.3	1.4	1	0.7	0.38	0.2	1.3	2.6	0.5	1.3	1.89	0.22	1
CD66 (a,c,d,e)	0.43	3.1	1.3	0.34	0.9	0.8	0.05	0.8	1.3	0.45	0.8	2	0.38	0.35	1.6
CD66b	0.53	0.3	0.4	0.3	1.4	0.9	2.76	0.5	1.4	0.14	0.6	0.9	0.13	0.41	1.4
CD66f	0.07	0.3	2.8	0.29	0.8	0.7	0.05	0.4	1.5	0.24	0.5	0.9	0.02	0.34	1.1
CD69	0	0.3	0.3	0.25	1.3	0.6	0.07	0.5	1	0.22	0.6	1	0.15	0.24	1.2
CD70	18.4	0.4	0.4	0.81	1.1	1.5	0.17	0.6	2.1	0.21	0.5	1.3	0.07	0.16	0.9
CD71	98	99.8	99.4	93	100	98.5	81.4	99.6	99.5	97.6	100	99.7	93	97	99.8
CD72	1.69	0.9	1	0.25	2.1	0.9	0.78	0.8	1.6	1.6	0.8	1.5	1.04	0.35	1.5
CD73	98.3	100	100	99.8	100	100	98.5	100	100	97.8	100	100	99.2	99.8	99.9
CD74	0.23	0.4	0.5	0.43	1.5	1.1	1.71	2.7	1.9	73.2	67	8.5	37.3	47.9	16.3
CD75	0.13	0.4	0.4	0.7	2	0.8	0.58	0.5	1.6	1.76	0.7	1.5	0.47	0.45	1.7
CD77	0.1	0.6	0.9	1.07	3.2	1.4	4.96	5.9	5.2	2.34	1.9	3.4	1.29	1.93	3.6
CD79b	0.03	0.6	0.9	1.17	1.9	1.3	1.78	1.4	2.6	0.29	1	3	0.68	0.67	3.5
CD80	0.16	0.3	0.4	0.28	1.1	0.6	0.25	2.7	3.2	4.17	5	3.4	0.72	2.95	3.1
CD81	98.3	99.9	99.9	99.9	99.9	99.9	98.6	99.8	99.7	100	99.9	99.9	98.3	99.5	99.8
CD83	1.88	0.6	0.8	1.07	0.9	0.9	1.39	0.7	1.8	1.62	0.9	1.4	1.77	0.32	2.1
CD84	0.13	0.3	0.2	0.27	0.8	0.8	0.12	0.5	1.4	0.14	0.5	1.2	0.12	0.39	1.1
CD85	0.23	0.2	41.8	0.28	1	0.6	0.09	0.7	1.3	0.27	0.9	1.5	0.13	0.16	1.3
CD86	0.26	0.3	0.5	0.29	1.8	1.2	0.07	0.4	0.7	0.17	0.4	1.2	0.05	0.18	0.6
CD87	0.13	0.2	0.4	0.36	2.6	3.2	0.09	0.4	2.6	1.99	10.8	9.5	0.08	1.44	8.3
CD88	0.15	0.4	0.2	0.18	1.2	1.7	15.8	0.2	1.6	0.3	5.7	5.8	0.08	0.73	5.1
CD89	0	0.2	0.2	0.23	1	1.1	0.15	0.2	0.9	0.12	0.5	2.4	0.07	0.24	1.2
CD90	99.6	99.8	100	99.6	99.9	100	98.3	99.9	99.9	96.6	99.3	99.9	98.6	96.5	99.8
CD91	80.4	99.5	99.2	63.1	98.7	84.5	57.9	94.2	91.2	95.8	96.3	90.7	64.3	90.6	95.8

Marker	PS2			PL			Selected FBS			Selected FBS +FGF2			Unselected FBS +FGF2		
	D191	D197	D199	D191	D197	D199	D191	D197	D199	D191	D197	D199	D191	D197	D199
CDW93	0.86	1	0.6	0.94	1.7	1.1	0.7	0.3	1.1	6.21	0.4	1.5	1.18	0.28	0.8
CD94	0	0.3	0.4	0.25	1	1.1	0.12	0.3	0.7	0.33	0.4	0.9	0.1	0.25	1
CD95	98.2	99.9	100	99	100	100	96.5	100	99.9	98.3	100	100	99.5	99.8	99.8
CD97	99.8	95.4	90.8	88.6	97.6	91.8	94.2	99.9	98.2	92.9	98.8	96	63.4	97.3	90.9
CD98	99.8	99.9	100	99.6	100	100	99.7	99.9	99.9	99.7	99.9	100	98.2	99.7	99.8
CD99	99.5	100	99.9	99.9	100	100	100	99.9	100	100	100	99.9	100	99.9	99.8
CD99R	99.3	76.6	98.6	83.7	92.9	69.3	94.4	98.8	86.6	99.3	92.9	95.5	98.3	73.8	75.9
CD100	0.43	0.3	0.3	1.2	0.6	1.2	0.4	0.2	0.3	2.36	0.1	1.4	0.97	0.22	0.4
CD102	19.5	15.4	7.2	7.38	33.3	13.5	32	73.6	43.1	49.3	69.5	31.8	29.7	66.5	26.5
CD103	0.33	0.3	0.4	0.29	1.3	1	0.34	0.3	1.1	1.38	0.3	1.5	0.42	0.22	0.8
CD105	99.4	100	99.9	99.6	99.9	100	99.2	99.9	99.9	98.5	99.8	100	99.2	99.8	100
CD106	3.08	13.5	6.9	6.24	67.3	29.7	68.8	96.4	80.1	82.2	94.2	85.3	90	85.6	67.1
CD107a	70	91.7	88.7	49.1	83.8	74.9	23.7	94.2	84.5	56.8	90.6	75.9	29.4	57.4	76.7
CD107b	23.6	35.1	32.7	8.59	32	24.4	10.8	68.2	36.7	21.6	41.2	28.2	3.39	8.16	30.6
CD108	99.5	99.9	99.9	98.2	99.9	100	83.8	100	96.4	92.4	99.9	99.1	76.9	99	97.2
CD109	19.6	69.5	82	4.22	96.5	97.3	23	99.1	91.3	95.8	99.1	98	93.8	97.2	86.9
CD112	2.98	6.1	10.7	0.63	2.4	1.7	0.3	5.1	1.9	1.96	2.2	3	1.24	0.29	1.1
CD114	0.1	0.3	0.5	0.3	1.5	0.9	0.02	0.2	0.7	0.19	0.3	1.2	0.07	0.26	0.9
CD116	56.5	0.5	0.6	0.66	2	1.4	69.7	0.4	1.3	40.8	0.3	1.7	11.5	0.47	1.5
CD117	0.53	0.4	0.3	0.32	1.7	1.1	41.2	0.2	0.8	1.25	0.3	1.1	0.08	0.26	0.7
CD118	0.29	0.2	0.4	0.23	1.4	1.1	0.09	0.4	1	0.15	0.3	1.3	0.13	0.2	0.7
CD119	30.3	72.8	76.2	2.54	74.2	50.4	3.28	91.5	75.7	43.6	90.2	87.2	12	45.4	48.2
CD120a	0.86	2.8	1.1	0.78	38.4	4	0.07	21.9	2.5	4.29	15.5	12	0.32	5.56	8.2
CD121a	3.73	6	2.2	0.79	38.5	10.2	0.58	40.6	11.6	24.1	17.1	31.9	11.9	16.8	11.2
CD121b	0.1	0.1	0.4	0.42	1	1	0.07	0.1	0.7	1.3	0.2	1.1	0.22	0.23	0.8
CD122	0.1	0.3	0.2	0.33	1.3	1	0.07	0.3	0.9	0.22	0.2	1.2	5.64	0.29	0.9

Marker	PS2			PL			Selected FBS			Selected FBS +FGF2			Unselected FBS +FGF2		
	D191	D197	D199	D191	D197	D199	D191	D197	D199	D191	D197	D199	D191	D197	D199
CD123	0.07	0.2	0.4	0.3	1.3	1.2	0.08	2.8	2.4	4.29	4.2	4.1	1.36	4.2	1.8
CD124	0.08	0.5	0.1	0.2	1.3	1	0.02	3.3	1.2	0.32	0.9	1.7	0.19	0.26	1.5
CD126	0.03	0.2	0.2	0.16	1.1	1.1	0.03	0.1	1	0.12	0.2	0.7	0.05	0.18	0.6
CD127	0.17	0.3	0.3	0.25	2.7	1.1	0.03	0.5	1.4	0.32	0.7	1.5	0.08	0.26	0.7
CD128b	0.2	2.1	0.5	0.31	0.5	0.8	93.8	0.2	0.4	0.15	0.2	4.4	0.96	0.31	1.6
CD130	56	77	71.6	6.18	83.5	49.1	97.7	87.9	78.8	75.1	93.2	96.5	30	65.9	76.3
CD134	0.92	0.8	1.1	0.27	1.4	1.2	0.56	0.4	1.3	0.32	0.2	1.2	0.4	0.16	1.1
CD135	0.19	0.3	0.3	0.2	1.7	0.9	71.7	0.2	0.9	0.44	0.1	1.6	0.08	0.29	0.7
CD137	0.08	0.3	0.4	0.25	2.2	0.9	0.08	0.4	0.9	0.17	0.3	1.7	0.1	0.2	0.6
CD137L	0.14	0.4	0.3	0.34	1.5	1.3	0.03	0.2	1.7	0.25	0.4	1.7	0.13	0.3	1
CD138	0.21	0.3	0.2	0.32	15.8	2.7	0.08	0.4	1.1	0.2	0.4	1.7	0.07	0.28	0.5
CD140a	88.9	54.4	24.3	0.33	19.3	3	80.2	99.3	97.1	97.7	99.9	99.2	96	98.9	96.3
CD140b	99.9	99.9	100	98.6	99.4	99.9	99.6	100	99.9	99.5	100	100	99.4	99.8	99.2
CD141	19.6	9.4	7.5	1.25	6	2.7	2.58	15.4	21.1	95	67.3	78	72.7	73.7	79
CD142	97.5	94.8	94.1	0.47	1.5	6.1	1.51	3	16.9	3.4	3.3	38.9	3.21	2.07	28.8
CD144	0.1	0.3	0.6	0.27	2	0.9	0.03	0.6	1.4	0.39	0.4	2.3	0.2	0.16	1.7
CD146	92	92.9	95.4	98.7	99.1	99.7	98.6	96.8	96.1	72.8	65.7	90.6	34.6	44.9	73.2
CD147	99.9	99.8	100	99.9	99.9	100	99.9	99.9	99.9	99.8	99.8	100	99.5	99.7	100
CD150	1.38	0.3	0.3	0.36	1.6	0.8	5.01	0.3	1.1	1.78	0.3	1.7	0.77	0.41	0.9
CD151	98.9	100	99.9	97.8	99.9	100	98.4	100	99.9	99.1	99.9	100	98.8	99.6	99.9
CD152	1.38	0.4	0.6	1.4	0.9	1.2	0.78	0.2	1.1	2.08	0.3	1.9	1.39	0.2	1.1
CD153	0.25	0.5	1.1	0.72	1	0.9	0.67	0.2	1.1	0.37	0.5	1.4	0.35	0.33	1.6
CD154	0.14	0.5	0.4	0.25	0.8	1.2	0.03	0.2	0.7	0.15	0.4	1.5	0.17	0.2	0.6
CD158a	0.07	0.2	0.3	0.35	0.7	1	0.05	0.3	0.7	0.13	0.2	1.5	0.1	0.28	0.8
CD158b	0.17	0.4	0.3	0.2	0.9	1.2	0.1	0.2	0.8	0.19	0.4	1.2	0.05	0.24	0.7
CD161	0.4	0.2	0.1	0.27	0.9	0.8	0.03	0.1	0.9	0.1	0.2	1.1	0.08	0.16	0.6

Marker	PS2			PL			Selected FBS			Selected FBS +FGF2			Unselected FBS +FGF2		
	D191	D197	D199	D191	D197	D199	D191	D197	D199	D191	D197	D199	D191	D197	D199
CD162	1.66	11.2	2.3	0.75	58.5	26.3	1.41	27.9	13.2	0.29	17.9	36.5	0.27	7.85	14.8
CD163	0.07	0.4	0.7	0.34	1.6	1	0	0.6	1.1	0.2	0.3	2	0.12	0.22	0.6
CD164	99.9	100	99.9	98.6	99.9	99.8	99.5	99.9	100	98.4	100	100	99.7	99.7	100
CD165	100	99.9	99.6	92.2	99.9	99.5	94.2	99.8	99.3	97.1	99.8	99.9	95.9	97.6	97.9
CD166	99.9	100	100	99.7	100	100	99.5	99.9	100	99.9	100	100	99.5	99.7	99.9
CD171	0.17	0.4	0.4	0.51	1.4	1.4	1.19	0.4	1.7	1.66	0.4	1.8	1.21	0.28	0.8
CD172b	0.11	0.2	0.1	0.13	0.9	0.8	0.03	0.3	0.8	0.22	0.3	1.7	0.12	0.22	0.8
CD177	0.07	0.1	0.5	0.24	0.9	0.9	0.07	0.2	1.1	0.2	0.3	1.5	0.07	0.16	0.8
CD178	0.2	0.2	0.1	0.16	1.1	1	0.07	0.2	1	0.15	0.2	1.3	0.07	0.14	0.7
CD180	0.1	0.5	0.4	0.87	0.6	1.3	0.6	0.5	1.1	0.74	0.3	1.9	0.2	0.27	0.8
CD181	99.4	81.6	59.4	15.7	91.3	43.2	17.2	76.4	59.2	47.4	68.7	76.5	48.1	60.5	72.3
CD183	8.55	0.9	0.4	0.87	1	1.4	0.74	0.5	1.9	0.89	0.5	1.8	0.98	0.61	1.8
CD184	0.1	0.4	0.9	0.31	0.7	1.3	0.1	0.5	1.3	0.22	0.2	1.5	0.1	0.26	0.9
CD193	0.07	0.7	1.6	0.67	1.9	1.7	0.29	0.4	2.3	0.19	0.5	3.3	0.24	0.47	2.7
CD195	0.23	0.3	0.7	0.98	1.3	1.3	0.62	0.4	1.4	0.31	0.5	2.5	0.17	0.53	1.8
CD196	0.5	0.8	1.9	0.53	1.6	1.3	0.19	0.5	2	0.31	0.6	2.5	0.28	0.7	2.1
CD197	0.2	0.4	0.9	0.87	1	1.1	0.76	0.4	1.1	0.87	0.6	1.9	1.49	0.37	1.4
CD200	6.16	20	23.3	1	3.4	6.1	9.48	10.8	34.6	37	52.9	80.6	46.9	28.9	70.4
CD205	1.55	1.3	1.4	0.47	2.1	3	4.15	5.3	8.2	52.9	20.4	33.7	53	12.8	17.6
CD206	0.08	0.7	0.2	0.25	1.1	1.1	0.1	0.4	0.9	1.24	0.2	1.7	0.03	0.27	0.4
CD209	1.16	0.4	1.2	1.7	1.2	1.4	1.84	0.6	1.7	0.54	0.5	2.3	0.74	0.61	2.3
CD220	0.1	0.8	0.4	0.88	18.7	13	0.15	48.9	13.8	3.78	16.3	29.3	1.11	1.24	8.9
CD221	99.8	89.2	88.6	25.4	80.6	62.1	5.87	96.4	79.5	32.8	69.2	88.6	12.3	19.5	47.5
CD226	1.09	0.4	0.6	0.23	0.8	1.3	0.19	0.4	1.3	0.5	0.2	1.2	0.17	0.31	1.1
CD227	32.9	32.1	37	54.2	69.8	70.8	69.9	80.2	81.1	75.5	90.6	91.8	84.8	78.4	66.6
CD229	0.75	0.4	0.4	0.44	1.4	0.8	0.26	0.5	1.2	1.55	0.6	2.5	0.66	0.33	1

Marker	PS2			PL			Selected FBS			Selected FBS +FGF2			Unselected FBS +FGF2		
	D191	D197	D199	D191	D197	D199	D191	D197	D199	D191	D197	D199	D191	D197	D199
CD231	0.23	0.5	0.6	0.38	1.7	1.3	0.04	0.4	1	0.26	0.9	2.6	0.12	0.47	1
CD235a	0.1	25.3	4.3	0.44	8.2	6.5	0.24	0.7	1.7	0.45	1.3	2.8	0.12	0.82	2.4
CD243	0.32	0.6	0.8	0.93	2.1	1.7	0.86	0.8	2.3	0.66	1.1	2.7	0.44	0.76	2.8
CD244	0.32	0.4	0.3	0.27	1.6	0.7	0.05	0.4	1.1	0.09	0.2	1.8	0.07	0.21	0.8
CD255	0.13	0.3	0.4	0.33	1.4	1.1	0.07	0.6	1	0.26	0.5	1.7	0.12	0.27	1
CD268	0.25	0.2	0.4	0.36	1.2	0.8	0.07	0.1	1	0.28	0.5	2	0.05	0.21	0.7
CD271	0.79	18.4	11.6	0.91	18.5	3.4	0.53	2.9	2.2	1.31	6.3	2.7	0.36	1.09	1.4
CD273	97.3	99.3	99.6	94.7	99.7	99.9	53.9	98.6	98.3	45.7	94.5	98.8	24.6	69.1	87.9
CD274	99.5	99.9	100	87.6	100	99.9	5.01	84.9	86.5	16.8	84.6	97.1	1.73	38.3	78.5
CD275	0.89	0.5	1.1	1.02	1.1	1.2	0.99	0.6	1.8	0.95	0.7	1.9	0.55	1.11	2
CD278	54.9	0.3	0.5	0.33	0.7	0.8	0.02	0.3	0.6	0.09	0.3	1.1	0.14	0.22	0.7
CD279	0	0.6	0.3	0.22	0.9	1	0.03	0.1	1.4	0.16	0.4	1.2	0.16	0.24	0.4
CD282	1.49	0.4	0.1	0.26	0.6	1	0.05	0.1	1.7	0.16	0.2	0.9	0.14	0.23	0.4
CD305	0.5	0.6	0.4	0.29	0.8	1.3	0.05	0.2	1.5	0.18	0.2	1.3	0.1	0.29	0.6
CD309	0.2	0.3	0	0.24	0.8	0.9	0.05	0.2	1.1	0.19	0.2	1.3	0.1	0.25	0.5
CD314	0.05	0.4	0.3	0.25	0.9	0.9	0.04	0.1	0.9	0.16	0.2	1.3	0.14	0.16	0.7
CD321	0.27	0.6	1	1.28	2	4.1	0.3	2.4	2.4	2.08	2.6	4.4	2.25	1.17	1.7
CD327	0.17	0.3	0	0.33	0.7	0.9	0.05	0.2	0.6	0.1	0.1	1.4	0.07	0.35	0.4
CD328	0.03	0.3	0.4	0.16	0.6	1	0.09	0.2	0.5	0.16	0.3	1.1	0.05	0.35	0.5
CD329	0.11	0.6	0.2	0.27	0.8	0.6	0.12	0.2	0.6	0.31	0.3	1.6	0.15	0.2	1
CD335	0	0.3	0.2	0.21	0.6	0.4	0	0.1	0.7	0.17	0.2	1.3	0.09	0.33	0.3
CD336	0.06	0.2	0.1	0.26	0.5	0.6	0.11	0.1	0.3	0.14	0.1	1	0.17	0.27	0.3
CD337	0.14	1.4	0.1	0.53	1.2	1.1	0.47	0.3	1.4	0.44	0.3	1.9	0.5	0.41	1.3
CD338	0.1	0.7	0	84.8	2.1	1.9	0.23	2.3	4.3	39.2	5.3	26.2	3.19	4.94	12.5
CD340	98	99.9	99.6	70.8	98.3	99.6	76.1	99.9	99.9	97.8	99.6	100	91.1	98.6	97.9
αβTCR	1.61	1.3	0.4	1.68	1.3	1.3	2.06	0.2	1.9	1.33	0.4	2	1.05	0.47	1.5

Marker	PS2			PL			Selected FBS			Selected FBS +FGF2			Unselected FBS +FGF2		
	D191	D197	D199	D191	D197	D199	D191	D197	D199	D191	D197	D199	D191	D197	D199
β2-microglobulin	97.4	100	97.6	99	100	100	97.9	99.9	99.9	99.1	99.8	99.9	98.9	99.5	99.5
BLTR-1	0.69	0.5	0.1	1.24	1.2	0.8	0.08	0.2	1	2.18	0.2	1.3	0.98	0.41	0.5
CLIP	0.07	0.3	0	0.25	0.9	1	0.05	0.2	1	0.5	0.3	1.8	0.1	0.21	0.5
CMRF-44	0.24	0.3	0.1	0.34	1.1	0.9	0.11	0.2	0.8	0.57	0.4	1.6	0.08	0.29	0.6
CMRF-56	0.27	0.5	0.5	0.41	0.5	0.8	0.39	0.1	0.6	35.3	11.3	2.5	18.1	12.2	4.2
EGFR	98.7	99.9	100	28.8	97.2	95.3	78.3	99.8	98.8	99	100	100	98.7	99.7	99.6
fMLP receptor	1.02	2.9	4.4	0.64	1	2.6	2.11	0.2	0.9	1.03	0.6	9.6	1.8	0.12	0.9
γδTCR	0.04	0.5	0.2	0.2	0.5	0.7	0.06	0.1	0.3	0.21	0.3	1.5	0.066	0.31	0.4
HPC	1.86	5.4	6.2	0.89	10.4	11.5	19.5	62.1	58	49.5	83.5	71.7	59	81.3	61
HLA-A,B,C	99.6	99.9	99.7	99.8	99.8	99.9	99.5	99.9	99.5	99.8	99.9	99.9	99.7	99.7	99.8
HLA-A2	0.13	0.5	4.9	0.71	0.7	1.1	0.74	0.2	1.6	0.65	0.3	1.4	1.37	0.39	0.6
HLA-DQ	64.6	93	89	38.6	51.9	56.1	40.9	46.1	54.6	85.7	61.2	43.7	52	57.5	57.5
HLA-DR	0.68	0.3	0.1	0.42	1.3	1.2	7.71	12.9	3	99.6	98.2	64.1	95.9	98.7	64
HLA-DR,DP,DQ	0.11	0.6	0.4	0.36	0.9	1.2	5.06	4.7	1.9	98.8	95.8	50.6	93.7	94.8	55.6
Invariant NK T	0.19	0.3	0.1	0.23	1.1	0.9	0.07	0.4	1.1	1.2	25.7	15	0.3	12.2	6.6
GD2	2.84	28.1	2.5	21.6	64.2	67.2	36.5	87.2	45	28.4	43.1	47.9	41.9	44.6	40.3
MIC A/B	99.2	63.5	99.6	99.2	4	100	95.7	2.6	99.8	97.3	3.7	99.9	83.3	0.33	99
NKB1	0.74	0.3	0	0.87	0.5	0.8	1.87	0.1	0.6	0.75	0.2	1.8	1.06	0.08	0.5
SSEA-1	98.3	86	75.7	0.22	1	0.9	0.1	0.1	0.5	0.29	0.2	1.4	0.17	0.16	0.5
SSEA-4	7.22	13.4	9.5	23.5	71	63.6	32	73.2	53.4	89.6	60.1	93.2	62.5	65.3	90.7
TRA-1-60	0	0.4	0.1	0.2	0.8	1.1	0.95	0.1	1.6	0.69	0.3	1.6	0.09	0.29	0.4
TRA-1-81	0.21	0.4	0	0.34	0.8	0.8	0.05	0.2	1.4	0.17	0.2	1.3	0.03	0.18	0.5
Vβ 23	0.01	0.7	0.1	0.4	1.1	0.9	0.17	0.2	1.2	0.22	0.4	2.2	0.15	0.24	0.8
Vβ 8	12.6	1.2	0	0.25	0.9	1.1	0.14	0.4	1.5	0.26	0.5	2.2	0.14	0.35	0.9
CD326	0.28	0.8	0.4	0.69	1.6	1.2	1.2	2.3	1.9	0.49	0.5	2.5	0.49	0.76	1.9

Marker	PS2			PL			Selected FBS			Selected FBS +FGF2			Unselected FBS +FGF2		
	D191	D197	D199	D191	D197	D199	D191	D197	D199	D191	D197	D199	D191	D197	D199
CD49f	35.2	44.6	39.9	29.5	96	98.7	60	38.8	71.9	82.3	54.3	72.6	49.6	40.3	70.7
CD104	0.07	0.7	0.1	0.11	1.3	1.5	0.64	0.3	0.8	0.67	0.4	2.3	0.07	0.37	0.8
CD120b	0.03	0.4	0	0.6	1.2	1.7	0.09	0.3	0.5	2.56	0.5	4	0.17	0.41	1.1
CD132	3.14	0.3	0.1	1.56	1.2	1.3	0.24	0.4	0.8	0.38	0.4	1.2	0.18	0.35	0.7
CD201	99.7	98.4	98.9	85.3	93.9	95.4	63.6	88.8	70.2	96.5	96.3	93.6	96.8	94.2	92.9
CD210	0.43	0.4	0.1	2.15	1	1.5	1.3	0.3	0.7	0.84	0.2	1.5	1.16	0.18	0.7
CD212	0.07	0.3	0.2	1.51	1.1	1.4	0.13	0.4	0.9	0.46	0.1	1.5	0.1	0.3	0.4
CD267	0.03	0.3	0.6	1.69	0.9	1.3	0.12	0.4	1.3	0.26	0.3	1.6	0.03	0.28	0.5
CD294	0.07	0.3	0.1	1.45	1	1.2	0.08	0.4	0.7	0.41	0.2	1.6	0.1	0.18	0.3
SSEA-3	0.07	0.4	0.2	1.66	1	1.3	0.31	0.2	0.8	3.37	0.2	1.1	1.42	0.2	0.3
CLA	0.62	0.2	0	1.76	0.9	1.5	0.53	0.2	0.5	0.54	0.2	1.6	0.48	0.16	0.4
Integrin β7	0.03	0.3	0.1	1.16	0.8	1	0.14	0.2	0.7	0.37	0.2	1.4	0.08	0.28	0.4
CD317	0.43	2.64	2.68	1.55	0.92	1.3	6.64	15.1	6.31	7.76	38.7	22.9	9.1	50.7	13.4
